# α‐Synuclein Pathology Spreads in a Midbrain–Hindbrain Assembloid Model

**DOI:** 10.1002/advs.202409040

**Published:** 2025-04-17

**Authors:** Gemma Gomez‐Giro, Daniela Frangenberg, Daniela Vega, Alise Zagare, Kyriaki Barmpa, Paul M. A. Antony, Graham Robertson, Rahman Sabahi‐Kaviani, Kristian Haendler, Nathalie Kruse, Florentia Papastefanaki, Rebecca Matsas, Malte Spielmann, Regina Luttge, Jens C. Schwamborn

**Affiliations:** ^1^ Developmental and Cellular Biology Luxembourg Centre for Systems Biomedicine University of Luxembourg Belvaux L‐4367 Luxembourg; ^2^ Bioimaging Platform Luxembourg Centre for Systems Biomedicine University of Luxembourg Belvaux L‐4367 Luxembourg; ^3^ Department of Mechanical Engineering Eindhoven University of Technology (TUE) Eindhoven 5612 AE The Netherlands; ^4^ Institute of Human Genetics Universitätsklinikum Schleswig–Holstein (UKSH) 23538 Lübeck Germany; ^5^ Human Embryonic and Induced Pluripotent Stem Cell Unit Hellenic Pasteur Institute Athens 11521 Greece

**Keywords:** α‐synuclein, brain organoid, in vitro disease modeling, Parkinson's disease

## Abstract

Understanding the progression of α‐synuclein pathology in neurodegenerative diseases such as Parkinson's disease (PD) is a longstanding challenge. Here, a novel midbrain–hindbrain‐assembloid model that recapitulates the spread of α‐synuclein pathology observed in PD patients, akin to Braak's hypothesis, is presented. Initially, the presence α‐synuclein pathology is demonstrated in the hindbrain organoids. Subsequently, sophisticated tissue engineering methods are employed to create midbrain–hindbrain assembloids. These assembloids allow investigation and description of the spreading of α‐synuclein pathology, as it progresses from the hindbrain components to the midbrain regions within the integrated structure. It is observed that an increase in α‐synuclein in the hindbrain can induce transfer of the pathology into the healthy midbrain, as well as cause changes at the synapse level. The presented model constitutes a robust in vitro platform for investigating the mechanisms underlying α‐synuclein spreading and disease progression, and holding potential for the screening of prospective therapeutics targeting the pathological propagation in PD and related synucleinopathies.

## Graphical abstract

A novel midbrain–hindbrain assembloid model demonstrates the spread of α‐synuclein pathology, a hallmark of Parkinson's disease, mimicking Braak's hypothesis. This model reveals how pathology propagates from the hindbrain to the midbrain, inducing synaptic changes and early signs of neuronal vulnerability. It offers an innovative platform for exploring neurodegenerative mechanisms and testing therapeutic interventions.

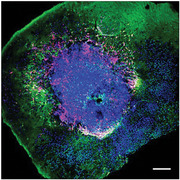

## 1 Introduction

According to the Braak's hypothesis for Parkinson's disease (PD), there are two main entry points for the pathology within the brain: the olfactory bulb and the lower brainstem. The pathology spreads from the olfactory structures to the amygdala and related areas, while pathology originating in the lower brainstem moves upward through vulnerable regions of the medulla, pons, and midbrain. This progression ultimately leads to the death of susceptible neuronal populations, including dopaminergic neurons (DNs) in the midbrain's “substantia nigra pars compacta” (SNpc).^[^ ^1^, ^2^ ^]^ A key pathological feature of PD and related synucleinopathies is the presence of α‐synuclein (α‐Syn) inclusions, which are the main component of Lewy bodies.^[^ ^3^ ^]^ Genetic factors play a significant role in PD development, with mutations in the *α‐Syn encoding* gene (SNCA)—including point mutations^[^ ^4^, ^5^, ^6^, ^7^, ^8^, ^9^, ^10^ ^]^ and gene copy multiplications^[^ ^11^ ^]^—linked to early‐onset familial PD with autosomal dominant inheritance. However, it is not yet clear which forms of α‐Syn‐mediated key processes in the disease dysfunction, such as aggregation, seeding propensity, or spreading. It has been shown that mutations affecting the structure and the folding of the protein,^[^ ^12^ ^]^ as well as α‐Syn post‐translational modifications, such as phosphorylation or acetylation, can modulate the equilibrium between physiological and pathological α‐Syn forms.^[^ ^13^ ^]^ Moreover, studies show that increased presence of wild‐type α‐Syn protein in biospecimens, such as blood and cerebrospinal fluid (CSF) Suppressor of mother against decapentaplegic, from patients harboring *SNCA* gene triplication influences the seeding activity and the deposition of aggregated forms into insoluble fractions.^[^ ^14^, ^15^ ^]^

The processes of α‐Syn seeding and aggregation have been observed in both laboratory cell cultures^[^ ^16^, ^17^ ^]^ and living experimental models.^[^ ^18^, ^19^ ^]^ Some of these studies have identified that distinct α‐Syn conformations have different seeding propensities and spreading capacities, which translate into different pathological phenotypes.^[^ ^20^ ^]^ Others have revealed the diverse mechanisms by which α‐Syn can transfer between cells^[^ ^21^, ^22^ ^]^ and propagate across different brain regions^[^ ^23^ ^]^ or even from the peripheral tissues into the central nervous system.^[^ ^24^ ^]^ The majority of these studies make use of overexpression systems, cell grafts, brain homogenates, or exogenous treatments with α‐Syn species,^[^ ^25^ ^]^ yet none of the models fully focus on the endogenous protein. More recently, key features of synucleinopathies have been modeled in more complex 3D organoid culture systems. For instance, human midbrain‐specific organoids derived from patients with an *SNCA* gene triplication exhibit elevated levels of α‐Syn and an increase in the protein phosphorylation at the serine residue 129 (pS129), associated with its aggregation and toxicity.^[^ ^26^, ^27^, ^28^ ^]^ However, these models only recapitulate the pathology in the midbrain.

Even though the complex circuitry of the structures comprised in the hindbrain coordinates fundamental functions, including heartbeat and respiration, locomotor activity, alertness, sleep, and wakefulness,^[^ ^29^ ^]^ human in vitro cell culture models of the hindbrain are very recent. Muguruma et al. generated 3D cerebellar cultures from embryonic stem cells resembling the early human cerebellum.^[^ ^30^ ^]^ Eura and collaborators designed a protocol for human brainstem organoid derivation, containing the midbrain, surrounding brainstem parts, and neural crest region.^[^ ^31^ ^]^ More recently, an alternative model for hindbrain‐fate organoids to generate serotonin‐enriched organoids was presented by Valiulahi and collaborators.^[^ ^32^ ^]^ These models provide great tools to investigate central nervous system development as well as disorders affecting brainstem structures. Building up on these previous efforts, here we developed a protocol to generate hindbrain organoids (HBO) using human‐induced pluripotent stem cells (hiPSC) and used it to recapitulate Braak's hypothesis for PD in vitro. We used a patient‐specific hiPSC cell line harboring the *SNCA* triplication, along with healthy controls and showed PD pathology in the hindbrain model. Going one step further, we generated healthy midbrain organoids (MO) and assembled them together with either healthy control or PD hindbrain organoids, to study the effect of α‐Syn pathology starting in the hindbrain over the healthy midbrain. The assembloid model suggests that increased α‐Syn in the hindbrain can spread to the healthy midbrain and induce α‐Syn pathology there, indicative of transfer of the pathology between both region‐specific organoids. Moreover, our results demonstrate that pathological α‐Syn, originating from the hindbrain, can contribute to early synaptic dysfunction, increasing the vulnerability of dopaminergic neurons to degeneration in the midbrain.

## 2 Results

### 2.1 Generation and Characterization of Human Hindbrain Organoids

**Figure 1:**
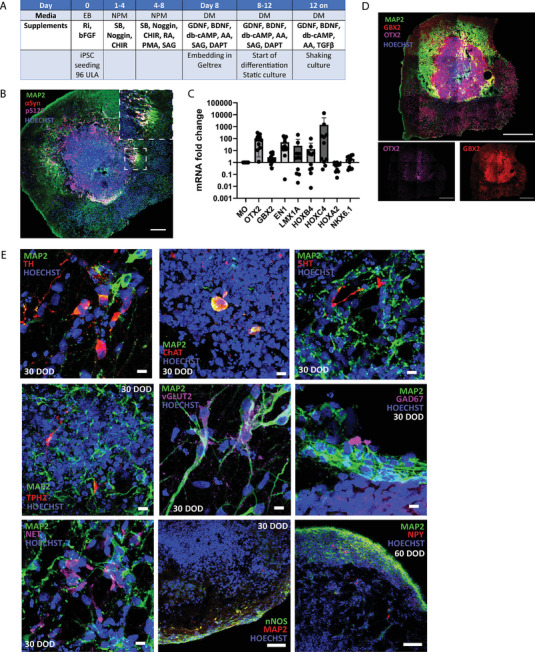
Generation and characterization of hindbrain organoids. A) Table outlining the protocol for the generation of hindbrain organoids. B) Representative immunostaining of an HBO 70 µm section at 30 days of organoid maturation, showing the presence of MAP2‐positive mature neurons, as well as neuronal markers α‐SYN and pS129. For better visualization, a zoomed area is offered (square). Scale bars = 200 µm. C) qRT‐PCR expression analysis for hindbrain markers expressed as fold change to their expression in the midbrain organoids at 30 days of organoid differentiation. HBO lines are pooled (Ctrl_1, Ctrl_2, and 3×SNCA) and MO line is Ctrl_2. Data represent the mean ± SD gene expression from organoids from three independent HBO and MO batches, *N* = 2–3 pooled organoids per line per RNA extraction. D) Representative immunostainings of 70 µm HBO sections at 30 days of organoid maturation, showing the presence of MAP2+ neurons, OTX2+ midbrain, and GBX2+ hindbrain precursor markers. Scale bars = 500 µm. E) Representative immunostainings of HBO sections at 30 days of organoid maturation, showing neuronal diversity by expression TH+ dopaminergic neurons, ChAT+ cholinergic neurons, 5‐HT+ and TPH2+ serotoninergic neurons, vesicular glutamate transporter 2 (vGLUT2)+ excitatory, and GAD67+ inhibitory neurons and NET+ noradrenergic neurons. Images taken at 63× magnification. Scale bars = 10 µm. Images of nNOS+ and NPY+ neurons were taken at 60 days of organoid maturation using 20× magnification. Scale bars = 100 µm. MAP2: microtubule associated protein 2; OTX2: orthodenticle Homeobox 2; GBX2: gastrulation brain Homeobox 2; TH: tyrosine hydroxylase; 5‐HT: 5‐hydroxytryptamine; TPH2: tryptophan hydroxylase 2; ChAT: choline O‐acetyltransferase; vGLUT2: vesicular glutamate transporter 2; GAD67: glutamate decarboxylase 1; NET: norepinephrine transporter; nNOS: neuronal nitric oxide synthase; and NPY: neuropeptide Y.

To explore the potential for modeling the early stages of PD pathology in the hindbrain, we generated HBO from hiPSC. The hindbrain represents one of the key subdivisions that emerge during neural tube development. The formation of these distinct regions is governed by complex morphogen gradients along both the anteroposterior and dorsoventral axes. Specifically, signaling pathways involving Wnt, retinoic acid (RA), and sonic hedgehog (Shh) play crucial roles in establishing these developmental axes, and its modulation is used in vitro to mimic developmental patterning and drive cellular regional identity.^[^ ^46^, ^47^ ^]^ Previous studies, which have attempted to achieve brainstem or hindbrain‐fate region‐specific organoids,^[^ ^31^, ^32^ ^]^ either avoided Shh and Wnt signaling in an effort to prevent ventral midbrain specification,^[^ ^31^ ^]^ or focused on ventral caudalization in order to generate 5‐hydroxytryptamine (5‐HT) neuron‐enriched organoids.^[^ ^32^ ^]^ Considering the diverse identity of the multiple nuclei and fiber tracts present in the hindbrain, we developed a strategy to generate organoids which would represent this miscellaneous hindbrain identity. To promote the differentiation of neural progenitor cells from hiPSC, we used dual inhibition of the suppressor of mother against decapentaplegic (SMAD) signaling through SB431542 and Noggin.^[^ ^48^ ^]^ At the same time, we applied CHIR99021, an activator of the canonical Wingless and Int‐1 (Wnt) signaling pathway, to guide the differentiation into caudalized neural fates. After 4 days, we introduced another caudalizing agent, RA, to induce the expression of *HOX* genes along the hindbrain axis. Additionally, to promote the appearance of ventral brainstem structures, we applied ventral agonists, smoothened agonist (SAG) and purmorphamine (PMA).^[^ ^46^ ^]^ After the initial prepatterning of the HBO, neuronal and glial differentiation was promoted by the addition of neurotrophic factors, such as brain‐derived neurotrophic factor (BNDF) and glial cell line‐derived neurotrophic factor (GDNF), and enhanced by the addition of DAPT (N‐[N‐(3, 5‐difluorophenacetyl)‐l‐alanyl]‐s‐phenylglycinet‐butyl ester) and TGFβ3 (transforming growth factor beta‐3) (**Figure** **1**A). At the end of the differentiation, HBO contained α‐Syn, a marker particularly important for synaptic function, showing the degree of maturity of the organoids (Figure 1B).

Analysis of 30 days old HBOs using quantitative reverse transcription polymerase chain reaction (qRT‐PCR) revealed elevated expression of multiple genes crucial for the establishment of midbrain and hindbrain structures. Orthodenticle Homeobox 2 (*OTX2*) and gastrulation brain Homeobox 2 (*GBX2*) genes, which are essential at the midbrain–hindbrain boundary,^[^ ^49^ ^]^ were both higher expressed in HBO compared to same‐aged MO. We also detected expression of ventral midbrain progenitor markers *LMX1A* and ventral caudal marker *NKX6.1*. Regarding *HOX* gene expression, we found higher expression of *HOXB4* and *HOXC4* in our HBO, whereas *HOXA2* was higher in the MO model, indicating differentiation of the HBO into hindbrain rhombomeric and spinal cord cervical regions^[^ ^50^ ^]^ (Figure 1C). The differences observed in expression patterns reflect the levels at this specific time point. It should be considered that these markers may peak at different times in differentiating midbrain and hindbrain organoids.

We further characterized the HBO model using immunofluorescence. At 30 days, we could detect neuronal precursors which are positive for OTX2 or GBX2, which indicated that the HBO model contains both midbrain and hindbrain components (Figure 1D). At this stage, microtubule associated protein 2 (MAP2) positive mature neurons were also abundant, and we were able to find different neuronal identities.^[^ ^51^ ^]^ As such, we identified tryptophan hydroxylase 2 (TPH2) or 5‐HT positive serotoninergic neurons, which, we suggest, arise from ventral hindbrain progenitors. Serotonergic neurons, which are primary components of the raphe nuclei, were observed. These nuclei are typically located in the basal plate of the pons and medulla.^[^ ^32^ ^]^ The presence of cholinergic neurons, identified by choline O‐acetyltransferase (ChAT) positivity, suggested the development of medulla populations.^[^ ^52^ ^]^ Noradrenergic neurons, marked by norepinephrine transporter (NET) positivity, were also detected. This may indicate the formation of structures akin to the “locus coeruleus,” a pontine nucleus that is a major source of norepinephrine in the brain.^[^ ^53^ ^]^ Interestingly, a subset of hindbrain norepinephrine neurons, known to project within the hindbrain and to forebrain areas including the hypothalamus, can co‐express norepinephrine Y (NPY).^[^ ^54^ ^]^ We indeed found NPY‐positive neurons in the HBO, after 60 days of differentiation. Additionally, vesicular glutamate transporter 1 (vGLUT1) excitatory and glutamate decarboxylase 1 (GAD67) inhibitory neurons were also found in the model. Lastly, we discovered some neurons were positive for neuronal nitric oxide synthase (nNOS), which is largely expressed in GABAergic interneurons^[^ ^55^ ^]^ (Figure 1E). Additionally, we found tyrosine hydroxylase (TH)‐positive dopaminergic neurons, which could indicate the presence of “nucleus tractus solitaries” (NTS) identity, where dopamine has a functional role modulating cardiorespiratory control,^[^ ^56^ ^]^ but also of midbrain “substantia nigra” identity, both important centers in the brainstem. The presence of all these cell types shows that the HBO model recapitulates the stunning neuronal diversity that characterizes the hindbrain, while still containing some remnants of midbrain identity. A positive Fontana–Masson staining at later stages (90 days of differentiation) indicated the presence of neuromelanin (Figure S1A, Supporting Information), which we attribute to the presence of the noradrenergic “locus coeruleus” neurons in the organoids.^[^ ^57^ ^]^ However, it is important to note that not every organoid exhibits this pigmentation at this time point in differentiation. The variability in pigmentation among organoids can be attributed to differences in neuronal differentiation and maturation processes across individual organoid cultures. One of the control lines (Ctrl_2), which was less pigmented in the Fontana–Masson staining, also showed consistently less NET protein expression, already at 30 days of differentiation (Figure S1B,C, Supporting Information). We quantified the staining for the different markers of neuronal cell types in the HBO model in all the cell lines used in this study at 30 and 60 days of differentiation, as well as the presence of glia markers, highlighted by the expression of glial fibrillary acidic protein (GFAP) and S100β positive cells. Despite some degree of variability observed between them, we demonstrate that all the lines were able to differentiate and mature into HBO. Noteworthy, one of the lines (3×SNCA) showed significant astrogliosis at day 60 of differentiation, compared to the other lines (Figure S1D–F, Supporting Information).

**Figure 2:**
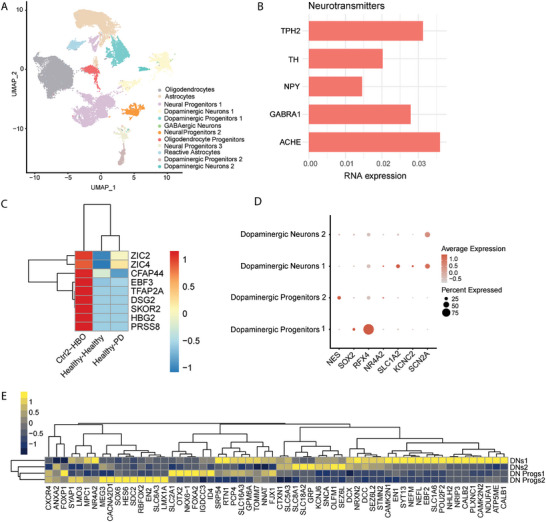
Single nuclei RNA sequencing characterization of hindbrain organoid populations. A) Unsupervised clustering of all cells from human hindbrain organoids generated using cell line Ctrl_2. B) Expression from different neurotransmitter genes within HBO. C) Hierarchical clustering of HBO sample (Ctrl_2 line) compared to healthy midbrain organoids based on expression of genes enriched in brainstem development. For this comparison, data from MO that had been assembled with either healthy (healthy–healthy) or 3×SNCA (healthy–PD) HBO were used. D) Dot plots showing a selection of genes that identify specifically different populations of dopaminergic neuronal precursors and neurons. E) Hierarchical clustering of all the genes that differentially classify the distinct populations of dopaminergic neurons (DNs) and progenitors (DN Progs) in the HBO sample (Ctrl_2 line).

To gain deeper insights into the cellular diversity and gene expression patterns in the HBO model, we employed single nuclei RNA sequencing (snRNAseq) on 60 days old control HBO (Ctrl_2 cell line). We analyzed the resulting data using dimensionality reduction techniques and applied unsupervised cell clustering through uniform manifold approximation and projection (UMAP), revealing 12 distinct cell cluster populations (**Figure** **2**A). Cellular identities were further determined based on the expression of canonical marker genes (Figure S2A, Supporting Information). We could identify different neuronal progenitor clusters, mature neuronal clusters, astrocyte and reactive astrocyte clusters, and oligodendrocyte clusters. Astrocytes were identified based on *ALDH1L1*, *GFAP*, and *S100B* gene expressions (Figure S2B, Supporting Information). The oligodendrocyte population was distinguished considering the expression of *MBP*, *MOG*, and *SOX10* (Figure S2C, Supporting Information). The matured neurons expressed pan‐neuronal markers *MAP2*, *MAPT*, *GAP43*, and *TUBB3* (Figure S3A, Supporting Information). The clustering highlighted mostly the presence of two major neuronal cell types: *GAD1/GAD2* expressing GABAergic neurons and two distinct populations of dopaminergic neurons, distinguished based on the expression of *TH, LMX1A*, *LMX1B, FOXA2*, and *EN1* genes (Figure S3B,C, Supporting Information). However, analysis of neurotransmitter expression confirmed the presence of serotoninergic (*TPH2*), *NPY*, and cholinergic (*ACHE*) neurons (Figure 2B). Noteworthy, when compared to MO, HBO expressed higher levels of hindbrain genes, such as *HOX* genes, *HOXA2/3/4* and *HOXC4/8/9* (Figure S3D, Supporting Information), and genes found to be enriched in brainstem, cerebellum, or cervical spinal cord, such as *ZIC2/ZIC4* ^[^ ^35^ ^]^ (Figure 2C). Moreover, we were able to differentiate two distinct subtypes of dopaminergic precursors, giving rise to two different populations of mature dopaminergic neurons, considering expression of early embryonic neural markers (*NES*,* SOX2*, and *RFX4*) and postmitotic dopaminergic neuron markers (*NR4A2*, *SLC18A2*, *KCNC2*, and *SCN2A*) (Figure 2D,E).^[^ ^58^ ^]^ Altogether, the snRNAseq data supported the presence of hindbrain identity in the here presented organoid model.

### 2.2 PD Patient‐Specific HBO Show α‐Synuclein Pathology

PD pathology, following the Braak's stages, initiates in the brainstem and the olfactory bulb and ascends, as the disease progresses, through the medulla, pons, midbrain, and basal forebrain, until it reaches the cerebral cortex.^[^ ^1^ ^,59]^ We aimed at recapitulating in vitro this disease progression from hindbrain to midbrain. Since the accumulation of α‐Syn is the main neuropathological hallmark of PD we decided to particularly focus on this characteristic. This also includes investigating the pathological α‐Syn variant with a pS129.^[^ ^26^ ^]^ For our analysis, we collectively compared PD patient‐specific HBO carrying an *SNCA* triplication (3×SNCA) to two controls lines (Ctrl_1 and Ctrl_2) derived from healthy individuals. Furthermore, the specificity of the α‐Syn antibodies was confirmed by using a genetically engineered line where the *SNCA* gene is deleted (SNCAKO).

**Figure 3:**
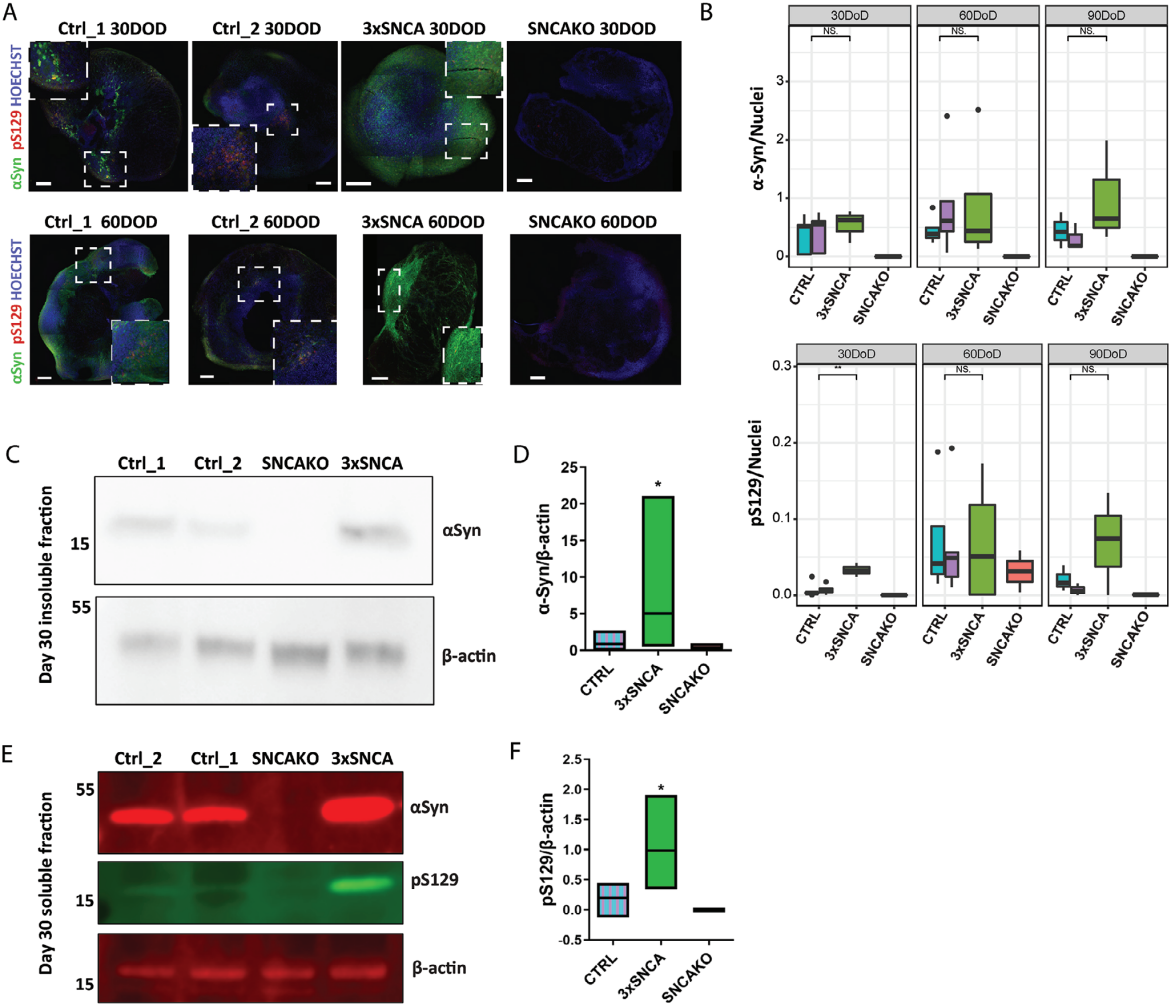
Increased pS129 α‐synuclein in 3×SNCA hindbrain organoids. A) Representative images of α‐Syn and pS129 staining in the HBO at 30 and 60 days of differentiation across all the lines. SNCAKO line is added to show specificity of the antibody. Magnified areas are delimited by a square drawn with a dashed line. Scale bars = 200 µm. B) Image analysis quantifications of α‐Syn and pS129 positive staining normalized to nuclei staining at 30, 60, and 90 days of HBO differentiation (DOD) in all the lines. Statistical significance was assessed by the Wilcox test: NS = Not significant, *p < 0.05. Data are represented as box plots with the line at the median and minimum and maximum value lines. Analysis was performed with organoid sections of at least three independent experiments, per line. C) Representative immunoblots of α‐Syn present in the insoluble fraction of control and 3×SNCA HBO lysates at 30 days of differentiation. SNCAKO cell lysate is added as a negative control. D) Quantification of insoluble α‐Syn at 30 days of organoid maturation showing the presence of more insoluble α‐Syn in the 3×SNCA HBO. Statistical significance by the Mann–Whitney test: *p = 0.0415. E) Representative immunoblot of soluble α‐Syn and pS129, showing an increase in 3×SNCA HBO at 30 days. F) Quantification of pS129 at 30 days of organoid differentiation. Statistical significance by the Mann–Whitney test: *p = 0.0476. Both for panels (D) and (F), data resulted from at least three independent organoid batches and are represented as box plots with the line at the median.

By immunofluorescence, we found that α‐Syn levels steadily increased from 30 to 90 days of differentiation in 3×SNCA HBO, while it decreased in control HBO between days 60 and 90, suggesting that α‐Syn levels are dynamic over time and different in the PD organoids. At 30 days of differentiation, we were able to detect a significant increase in phosphorylated α‐Syn (pS129), post‐translational modification suggested to modulate α‐Syn aggregation propensity. The levels continued to rise at the later time points, although the difference became not significant (**Figure** **3**A,B), probably due to the evident fluctuation of α‐Syn levels. The positive signal for pS129 was also found significantly in co‐localization with thioflavin S (ThioS) in 3×SNCA HBO, suggesting that insoluble α‐Syn is present early in the brainstem HBO model (Figure S4A,B, Supporting Information). To confirm that, we performed a sequential protein extraction to detect soluble and insoluble α‐Syn. Immunoblotting of the extracts showed significantly increased levels of α‐Syn in the insoluble fraction of 3×SNCA HBO at 30 days of organoid growth (Figure 3C,D). The levels of pS129 were also significantly increased in the soluble fraction (Figure 3E,F). Additionally, the 3×SNCA HBO also presented greater extracellular release of α‐Syn into the supernatant at this stage of differentiation as assessed by dot blot (Figure S4C,D, Supporting Information). Overall, our findings suggest that the 3×SNCA HBO are able to model the accumulation and aggregation of α‐Syn and its phosphorylated form in the brainstem. Together with α‐Syn immunoreactive aggregates, progressive neuronal loss in selected brain regions, such as the *SNpc*, the ventral tegmental area, or the cholinergic pedunculopontine nucleus, is also a neuropathological feature of PD.^[^ ^60^ ^]^ Yet, we did not observe any significant loss of a specific neuronal population in 3×SNCA HBO from day 30 to day 60 of differentiation (Figure S1F, Supporting Information), including the dopaminergic TH‐positive neuronal population (Figure S4E,F, Supporting Information). However, when we performed a deeper characterization of the presence of α‐Syn in the different neurons of the HBO, we found significantly higher α‐Syn in certain neuronal cell types and at different time points in the 3×HBO, compared to the control lines. α‐Syn was found higher in colocalization with NET‐positive neurons and GFAP‐positive glial cells at 30 days of differentiation, with TH‐positive neurons both at 30 and 60 days of differentiation and at the later time point in serotonin‐positive neurons (Figure S5A–J, Supporting Information).

### 2.3 Generation and Characterization of Midbrain–Hindbrain Assembloids

**Figure 4:**
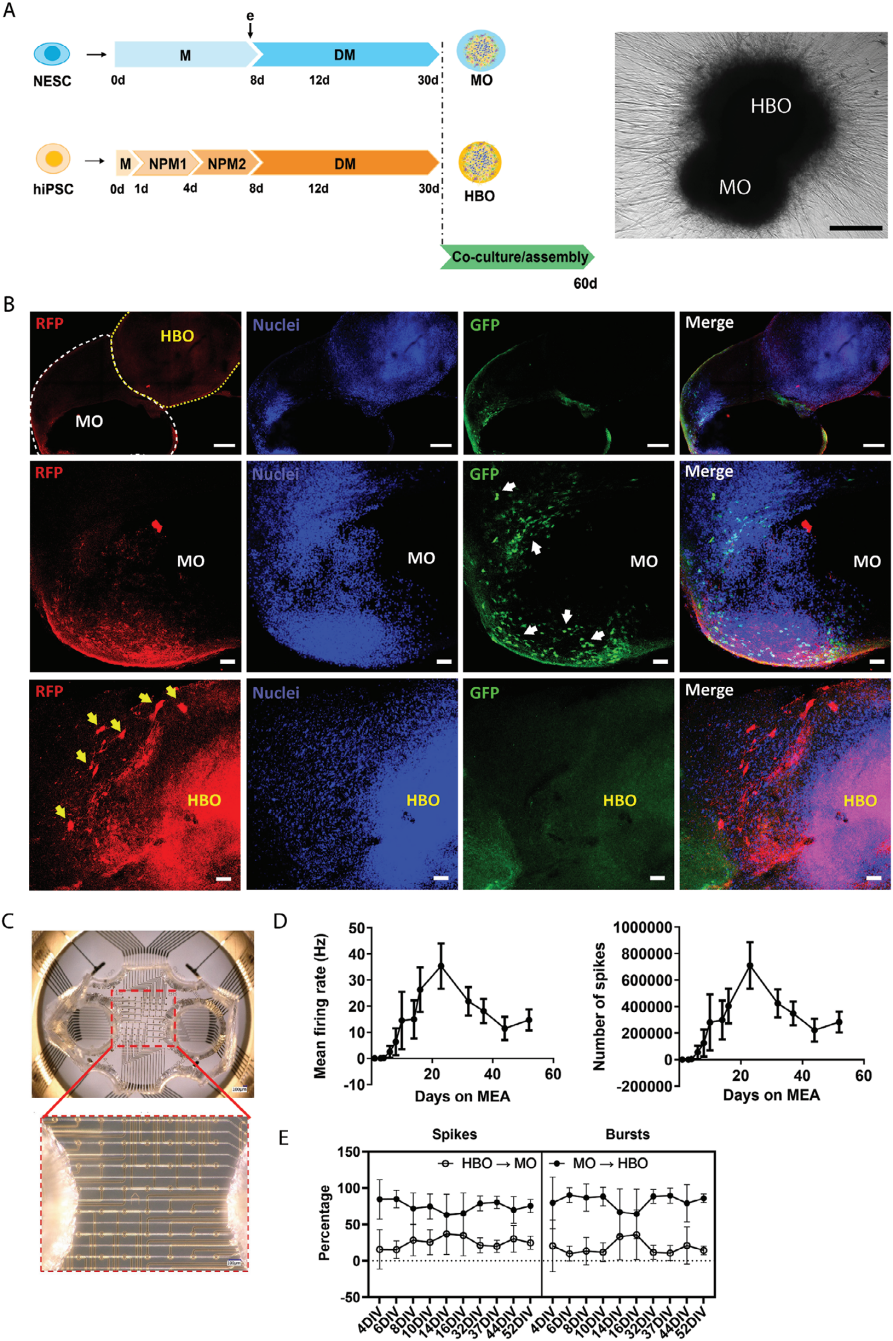
Generation and characterization of midbrain–hindbrain assembloids. A) Diagram representing protocols for midbrain organoid (MO) and hindbrain organoid (HBO) generation and time point for assembloid formation. Bright‐field picture of an assembloid 16 days after co‐culture. Scale bars = 500 µm. (legend: days (d), embedding (e), maintenance (M), NPM (neural patterning medium), DM (differentiation medium). B) Confocal images of 70 µm assembloid sections after retrograde vial tracing experiments for assessment of viral GFP and RFP expressions. Hoechst dye was added to stain nuclei. In the upper panel, a tile scan of the entire assembloid (20×) is presented. Dashed yellow lines highlight the HBO part of the assembloid in the section. White dashed line delineates MO part of the assembloid. Scale bars = 200 µm. Middle and bottom panels represent 20× snap pictures of regions within the MO (middle) or HBO (bottom) where we detected GFP‐infected cells (on the MO side) and RFP positive cells (on the HBO) highlighting synaptic connections exist between the two organoids comprising the assembloid. We highlighted some of the cells that we consider positive with either white (MO) or yellow (HBO) arrows. Scale bars = 50 µm. All images are maximum intensity projections from a *z*‐stack acquisition. C) Aligning the PDMS microtunnel device (MD) with the MEA substrate plate (Axion), ensuring proper alignment of the tunnels with the electrode array. Scale bars = 100 µm. D) Time course of mean firing rate and number of spikes of assembloids on the MD MEA over 50 days of assembly on the MEA. Both MO and HBO in this case were derived from the control cell line (Ctrl_2). E) Representation of the percentage of directionality of the electric signals (spikes and bursts) between MO and HBO on the MD MEA, highlighting bidirectional communication. The data shown in panels (D) and (E) correspond to the mean ± SD, and the analysis was performed for two independent MO and HBO batches, with three organoids per batch. In the same experiment data with healthy MO and PD HBO were collected, but only the control data are shown in this figure.

To model the spreading of α‐Syn pathology from the hindbrain to the midbrain, we combined hindbrain organoids together with midbrain organoids,^[^ ^33^ ^]^ creating an assembloid model. HBO and MO were initially generated separately and cultured independently for 30 days. Then, they were assembled and maintained together until reaching day 60 of cultures (**Figure** **4**A). To characterize the presence and specificity of HBO‐derived neuronal projections toward the MO, we used a retrograde rabies tracing approach.^[^ ^60^ ^]^ We separately infected MO with an EnvA receptor (TVA) expressing lentivirus tagged with green fluorescent protein (GFP) and carrying the envelope spike glycoprotein (GP) of the rabies virus required for virus *trans*‐synaptic spread (LV–GP–TVA–GFP).^[^ ^36^ ^]^ After 7 days of infection, we added the HBO and transduced the whole assembloid with a G‐deleted (ΔG) recombinant rabies viral vector, pseudotyped with the envelope protein (EnvA) which selectively binds to the TVA receptor and tagged with red fluorescent protein (RFP) (RBV–ΔG–EnvA–RFP).^[^ ^38^ ^]^ Assembloids were further cultured for 30 days, after which samples were fixed, sectioned, and analyzed using a confocal microscope. We predicted that initially infected MO neurons would express GFP from the LV–GP–TVA–GFP, and presynaptic HBO neurons connected to them would be RFP positive, due to the selective retrograde transmission of rabies virus.^[^ ^61^, ^62^ ^]^ Accordingly, we were able to find GFP‐positive nuclei in the MO region of the assembloids, while RFP labeled cells were found in the HBO region of the assembloid (Figure 4B). It was also possible to find co‐expression of both markers in the case of double‐transduced cells, which mark the starter population (Figure S6B, Supporting Information) and we were able to confirm that the rabies virus spread to presynaptic neurons, by looking at co‐expression of the RFP‐positive cells and the neuronal marker MAP2 on the HBO side of one of the infected assembloids (Figure S6C, Supporting Information). Our findings highlighted the presence of active synaptic connections between the two organoids in the assembloid.

To validate that the assembloid model exhibited functional neuronal connections, we performed microelectrode array (MEA) measurements. To understand the directionality of the signals between the different regionalized organoids, we decided to confine each organoid (one MO and one HBO) into a well of a two‐compartment customized polydimethylsiloxane (PDMS) device, connected by microtunnel devices (MDs) which were aligned over the planar electrode array of a 12‐well MEA plate (Figure 4C; Figure S6D–F, Supporting Information). This configuration allowed for the long‐term culturing of the organoids on the MEA plates, the recording of spontaneous activity in the axons confined inside the tunnels connecting the organoids and improved the signal‐to‐noise ratio, compared to conventional 2D MEA used with 3D organoid cultures.^[^ ^63^ ^]^ Over the days in vitro (DIV), we saw a fluctuation in the signals, with an initial pronounced increase in the number of spikes and the mean firing rate until ≈21 DIVs, followed by a decrease during the following days (Figure 4D). Taking a closer look to the directionality of the signals, we saw that around 80% of the spikes and bursts were initiated at the MO site, whereas about 20% were coming from the HBO site (Figure 4E), confirming a bidirectional communication of the axons between the different organoids.

### 2.4 α‐Synuclein Pathology Spreads from Hindbrain Organoids toward Midbrain Organoids

**Figure 5:**
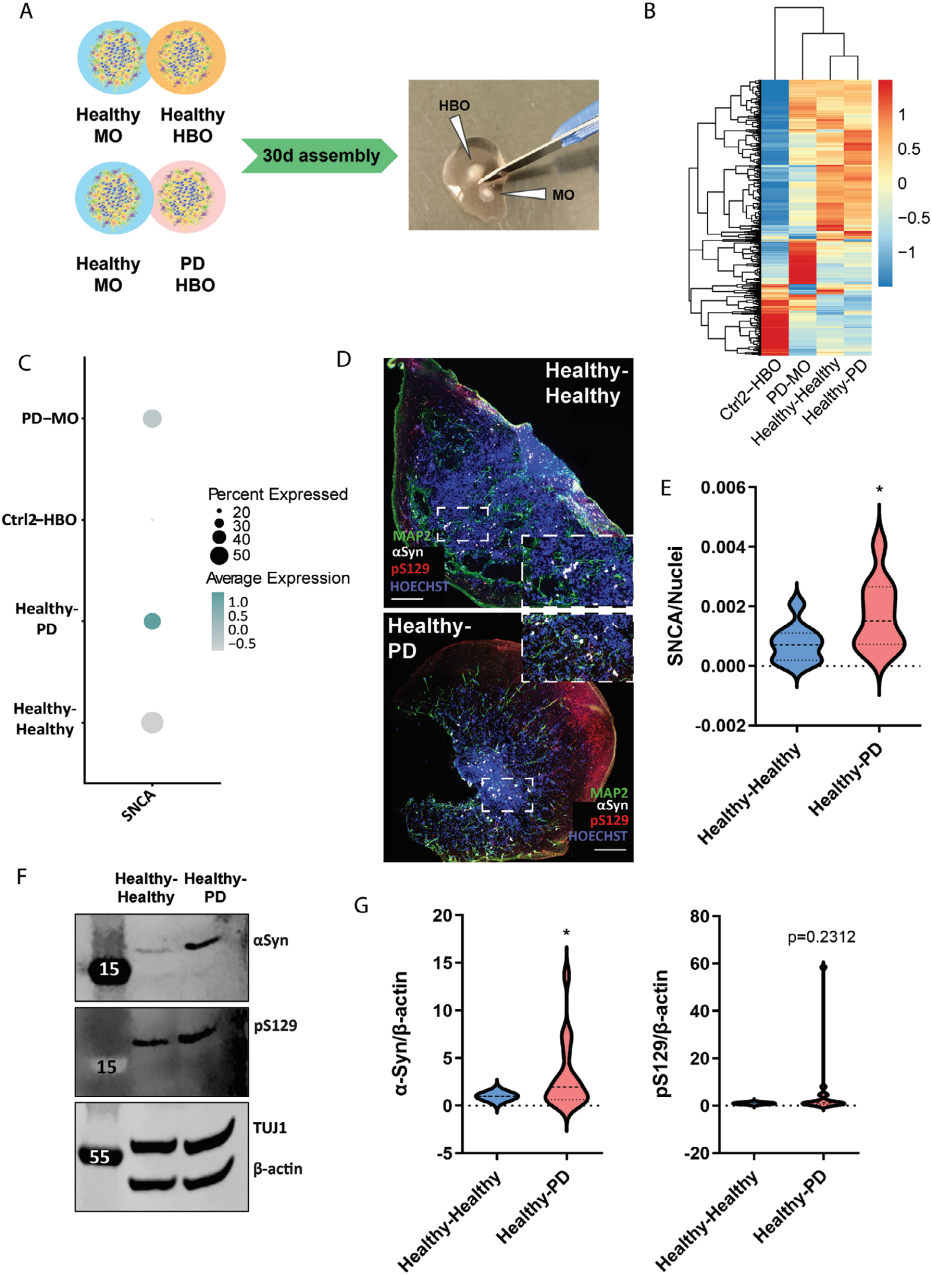
Healthy MO show an increase in α‐Syn upon assembly with PD HBO. A) Diagram showing the generation of different assembloids and the dissection of the samples after 30 days of assembly. B) Heatmap showing differential clustering of the assembloid samples separating from the HBO sample based on their gene expression profiles. C) Dot plot highlighting the expression of α‐Syn in the different models, showing higher expression in the MO upon assembly with 3×SNCA HBO. D) Representative confocal images of α‐Syn and pS129 staining in 70 µm sections of MO after assembly. A zoomed‐in region is offered for better visualization, inside the dashed area. E) Image analysis quantification shows increased α‐Syn staining levels on MO after assembly with 3×SNCA HBO. Data resulted from at least four independent organoid batches. Statistical significance by the unpaired *t*‐test: *p < 0.05. F) Representative immunoblots for α‐Syn and pS129 in MO upon assembly. G) Quantification of immunoblots for α‐Syn and pS129 showing the fold increase between the MO assembled with control HBO (values set to 1) and the MO assembled with 3×SNCA HBO. Data resulted from at least three independent organoid batches. Statistical significance assessed by one sample *t*‐test: *p < 0.05 for α‐Syn. For panels (E) and (G), data are shown as violin plots, where the dashed line represents the median and the dotted line, the quartiles.

Recent studies have shown that pathological α‐Syn species can transfer between multiple cell types, such as from neuron to neuron^[^ ^18^, ^21^ ^]^ or between microglia cells,^[^ ^22^ ^]^ suggesting that the pathology can potentially spread within different brain regions and between the brain and peripheral tissues. After finding α‐Syn pathology in the PD 3×SNCA HBO, we wanted to evaluate its potential spread from the hindbrain model to a healthy midbrain model. To simulate this, we generated interindividual assembloids where we had an MO and either a healthy or a diseased HBO. In both models, the healthy line used was the Ctrl_2 line, which was age and gender matched to the 3×SNCA PD line. In order to discriminate solely the effect on the MO, we first assembled the organoids and cultured them together for a month, after which we dissected them and postprocessed the MO component of the assembloids (**Figure** **5**A). To determine which organoid was the MO and which the HBO, we relied on both size and morphological features. hiPSC‐derived organoids (in this case, HBO) were significantly bigger than MO already a few days after the embedding (Figure S6A, Supporting Information) and exhibited a higher variability in morphology, which is likely due to their greater diversity in terms of cellular composition that pluripotent cells offer, as opposed to the more defined lineage commitment of the MO's starting population.

We performed snRNAseq for the MO in both assembloid conditions, namely, healthy midbrain–healthy hindbrain (H–H) and healthy midbrain–PD hindbrain (H–PD) and compared them to PD MO alone. Hierarchical clustering of all samples revealed that the healthy MO component within the assembloids exhibited a consistent gene expression pattern, irrespective of its association with healthy or PD HBO. Intriguingly, both MO samples displayed a transcriptomic signature more closely resembling that of a PD MO, while the HBO sample clustered separately (Figure 5B), underscoring its enriched diversity of cell types and reflecting the maintenance of the region‐specific identity despite the co‐culture. Additionally, we quantified the relative proportion of cells in each cluster per model, showing very similar amount of neuronal (most prominently dopaminergic neurons) and non‐neuronal (predominantly astrocytes) proportions in the MO which were co‐cultured with an HBO (Figure S7A and Table S4, Supporting Information). Interestingly, when we looked at the expression of α‐Syn, we found an increased *SNCA* expression in the MO that had been in contact with the PD HBO, in contrast to the one that had been assembled together with a healthy HBO or the PD MO alone (Figure 5C). To understand if this increase in *SNCA* expression translated into an increase at the protein level, we performed immunofluorescence in MO sections after assembly, which showed a significant increase in α‐Syn in the healthy MO upon assembly with a PD HBO (Figure 5D,E). At the evaluated time point, there was also a tendency to an increase in α‐Syn phosphorylation (pS129) (Figure S7B,C, Supporting Information). We evaluated if this difference was specific to the assembloid cultures, by comparing the α‐Syn levels of healthy MO at the time point before assembly (D30) and at the final time point after assembly (D60), where we saw no significant increase of α‐Syn (Figure S7D, Supporting Information). Additionally, western blot analysis showed a threefold increase on average in the levels of α‐Syn and phosphorylated α‐Syn (pS129) in the MO from the H–PD assembloids, compared to the H–H combination, although not significant for pS129 due to variation (Figure 5F,G). To gain deeper insight into α‐Syn distribution patterns per cell types, we checked the *SNCA* expression across the different clusters and models on the snRNAseq datasets. We found *SNCA* to be significantly upregulated in astrocytes, neural progenitors, GABAergic neurons, and dopaminergic precursor cluster 2 in the MO that had been exposed to the PD HBO (Figure S7E, Supporting Information), suggesting selective vulnerability of specific cell types in the midbrain to α‐Syn upregulation. To clarify if the diseased environment of the PD HBO alone could replicate the increase in α‐Syn levels, we cultured healthy MO with a 1:1 ratio of fresh and spent media coming from either healthy control or PD HBO cultures. α‐Syn levels, but not pS129, significantly increased in healthy MO exposed to PD conditioned media, compared to MO that received healthy HBO media (Figure S7F,G, Supporting Information), indicating that α‐Syn transport in its various forms does not only occur through direct cellular connection, but it is also taken up from the media. However, this might not be sufficient to induce the full pathology.

**Figure 6:**
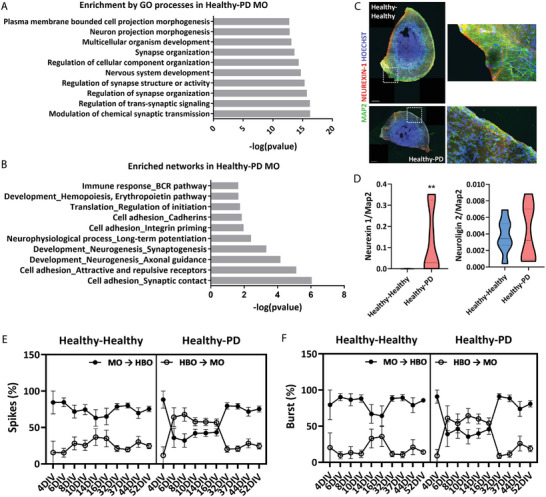
Healthy MO show synapse dysregulation upon assembly with PD HBO. A) Graph showing most enriched gene ontology (GO) processes among the differentially expressed genes between healthy–healthy and healthy–PD assembloids. B) Graph showing most enriched networks among the differentially expressed genes between healthy–healthy and healthy–PD assembloids. C) Representative images of synaptic marker Neurexin‐1 in MO after assembly with either healthy or PD HBO. On the right‐hand side of every picture, a zoomed‐out area is shown, corresponding to the designated area (squared) on the overview picture. Scale bars = 200 µm. D) Image analysis quantification of synaptic markers Neurexin‐1 and Neuroligin‐2 in MO sections after assembly. Data were collected from one section per organoid of at least five independent organoid batches. Data are shown as truncated violin plots, where the dashed line represents the median and the dotted line, the quartiles. Statistical significance assessed by the Mann–Whitney test: **p < 0.01 for Neurexin‐1. E,F) Representation of the percentage of directionality of the electric signals (spikes and bursts) between MO and HBO of the different assembloid conditions on the MD MEA, highlighting changes in the bidirectional communication on the healthy–PD condition. Please note that the healthy–healthy dataset is the same as previously shown in Figure 4, and the healthy–PD condition is added here for comparison. The data collection for the MD MEA experiment were performed for two independent MO and HBO batches, with three organoids per batch, which were placed within the same Axion plate. Data are represented as mean ± SD.

α‐synuclein is a synaptic protein and its pathological increase might lead to a dysregulation in the synapse.^[^ ^64^ ^]^ Analysis of the differentially expressed genes (DEGs) of the MO after assembly with healthy or PD HBO revealed an enrichment in gene ontology (GO) processes related to the structure of the synapse, and its organization and activity. These, together with nervous system development, were also the most enriched networks in the MO upon assembly with 3×SNCA HBO (**Figure** **6**A,B). Most genes integral to the synapse and its function were found upregulated (Figure S8A, Supporting Information). Neurexin‐1/Neuroligin‐2 complex plays a multifaceted role in organizing the synapse and regulating synaptic function, influencing synaptic transmission, and contributing to the overall balance of neural network activity.^[^ ^65^ ^]^ We found presynaptic marker Neurexin‐1 significantly higher in healthy MO upon assembly with PD HBO, while no significant difference was observed in postsynaptic marker Neuroligin‐2 (Figure 6C,D), confirming some changes in the organization of the synapse. We also assessed pre‐ and postsynaptic proteins synaptophysin and PSD‐95, respectively (Figure S8B,C, Supporting Information). To see if synaptic alterations would translate into changes in electrophysiological activity, we checked on the MEA how different the bidirectional communication was between H–H assembloids (Figure 4E), compared to the H–PD assembloids. Despite similarities in the number of spikes and mean firing rate between H–PD and H–H samples, there were instances where H–PD samples exhibited lower general activity, particularly in certain DIV (Figure S8D, Supporting Information). Analyzing the directionality of the signals, we noticed that there was a shift in communication directionality for some DIV, where spikes and bursts were more initiated at the HBO site (60%) and not at the MO site (40%) (Figure 6E,F), contrary to what we had seen before in the H–H assembloid (Figure 4E). However, the pattern returned to the values of the H–H situation after 32 DIV. Altogether our data suggest that there is a transfer of α‐Syn pathology from the hindbrain to the midbrain in the assembloid model, and that this excess can very early (30 days of assembly) cause alterations in the healthy midbrain tissue, starting at the synapse level on a first instance, probably before revealing a loss of the dopaminergic neuronal population.

## 3 Discussion

α‐synuclein immunoreactive aggregations and progressive neuronal loss in selected brain regions are characteristic neuropathological hallmarks of synucleinopathies, including PD and Lewy body dementia (DLB). These inclusions have been found in brainstem nuclei and nerve fiber tracts of PD and DLB patients,^[^ ^2^ ^]^ contributing to the hypothesis of α‐Syn pathology starting first in the hindbrain before spreading into the midbrain and other cortical regions.^[^ ^1^ ^]^ In this study, we present an hiPSC‐derived in vitro model of the hindbrain which allows the investigation of α‐Syn pathology in this region and can recapitulate the spread of the pathology into the midbrain by combining the two region‐specific organoids into an assembloid model. We first use healthy control lines to perform an in‐depth characterization of the hindbrain model. Anatomically, the hindbrain contains the pons, the cerebellum, and the medulla. These structures are directly connected rostrally with higher brain regions and are caudally continued by the spinal cord, expanding the connectome to peripheral regions. Hence, the nuclei contained in the hindbrain present an enormous diversity of neurons.^[^ ^29^ ^]^ These diversity is recapitulated in the HBO model by the presence of miscellaneous neuronal identities, such as serotoninergic, cholinergic, GABAergic, or noradrenergic, among others, highlighting the existence of neuronal populations from the pons and the medulla.^[^ ^52^, ^53^ ^]^ Accordingly, the gene expression pattern found high expression of markers associated to hindbrain development, such as *HOX* genes^[^ ^29^ ^]^ and genes found to be enriched in brainstem, cerebellum, or cervical spinal cord.^[^ ^35^ ^]^ Yet our study observed discrepancies in HOXA2 gene expression between qRT‐PCR and snRNAseq data. Specifically, qRT‐PCR data from 30 day differentiated organoids showed different expression patterns compared to snRNAseq data from 60 day differentiated organoids. This difference likely reflects the distinct stages of maturity, with snRNAseq capturing more detailed expression profiles within specific cell populations. Interestingly, the HBO also contained dopaminergic neurons, suggesting the presence of midbrain neuronal population. This is in line with other protocols attempting to reach hindbrain identity in hiPSC‐derived organoids,^[^ ^31^ ^]^ as opposed to organoids derived from prepatterned stem cells. However, compared to midbrain organoids generated from a precommitted stem cell population,^[^ ^34^, ^66^ ^]^ the HBO at 60 days of differentiation exhibited two distinct populations of dopaminergic precursors and dopaminergic neurons, showing that, even though there might be less dopaminergic neurons in the HBO model, their specification seems to be enhanced with this protocol. These data suggest that the model recapitulates the hindbrain in vitro and contains midbrain remnants, highlighting its aptness to study brainstem disorders.

In order to investigate the brainstem affection on PD patients, we generated HBO from an *SNCA* gene triplication patient. The 3×SNCA HBO showed significantly higher levels of insoluble α‐Syn, as well as an increased extracellular release. They also displayed significantly higher presence of its phosphorylated form, pS129, modification associated with increased aggregation capacity. Phosphorylated α‐Syn was found in co‐localization with ThioS staining, which labels amyloidogenic β‐sheet fibrils. Our findings are in line with other studies which reported increased thioflavin‐positive α‐Syn in 3×SNCA patient‐derived neurons^[^ ^67^ ^]^ and in MO carrying glucocerebrosidase and α‐Syn mutations.^[^ ^68^ ^]^ When we compared same‐aged MO and HBO, only HBO at 30 days of differentiation showed signs of α‐Syn pathology, recapitulating in vitro the start of the pathology in the brainstem. However, we observed that this difference in α‐Syn levels was not maintained at later time points, suggesting that α‐Syn levels fluctuate along the organoid differentiation. This decrease in α‐Syn accumulation over time was also seen in 3×SNCA patient‐derived dopaminergic neurons by day 49 of differentiation.^[^ ^69^ ^]^

It has been reported that overexpression of α‐Syn can initiate its long‐distance brain transfer and cause longlasting pathological changes along its trajectory.^[^ ^18^, ^70^ ^]^ In order to study potential spread of α‐Syn pathology, we built an assembloid containing MO as midbrain model and HBO as hindbrain model. As a combination of multiple region‐specific organoids, assembloids can capture intertissue cellular interactions and mechanisms happening through these interactions, such as spreading, at a higher level of complexity. The organoids in the assembloid model were able to form functional connections, recapitulating what has been seen before in other published assembloid models.^[^ ^35^ ^]^ By making healthy–PD assembloid combinations, we aimed to study the effect of α‐Syn overexpression in the brainstem toward an unaffected midbrain. Dissecting the different assembloid components after the assembly period allowed for a more specific analysis of the contribution of a healthy or PD hindbrain toward the midbrain compartment. To separate both organoids, we relied on size and morphological features. While this process works robustly, Dulbecco's Modified Eagle Medium the use of fluorescent‐tagged lines would further optimize this procedure. With this model, we were able to observe that even a mild α‐Syn accumulation in the 3×SNCA HBO was able to increase α‐Syn expression in the healthy MO, and in specific cell types, suggesting a potential starting level of selective vulnerability. Yet the increase in intrinsic expression does not necessarily demonstrate that it is due to transfer of aggregates, but it could be caused by the exposure to a PD environment without the need for a seeding effect. More efforts would need to be made to understand if spreading is indeed occurring or not.

Moreover, we found an increased presence of α‐Syn protein in the MO upon assembly with 3×SNCA HBO, not observed by the natural aging in culture of the MO, suggesting protein transfer between the organoids. Several mechanisms have been proposed by which α‐Syn can be transferred between cells, which include exocytosis, release through compromised cell membranes, exosomes, or tunneling nanotubes, among others.^[^ ^71^ ^]^ As we observed an increased release of α‐Syn in the media of 3×SNCA HBO, we checked if these α‐Syn could also be taken up by midbrain organoids by exposing healthy MO to spent media of healthy and PD HBO. Culturing MO with PD conditioned media also increased the levels of α‐Syn in the healthy MO, indicating that multiple mechanisms are involved in the transfer of α‐Syn in this model. Often, the increase we observed in α‐Syn did not necessarily correlate with higher pS129, suggesting that phosphorylation levels may be regulated independently of total α‐Syn levels. Studies have shown that pS129 levels can change rapidly in response to stimuli, such as neuronal activity.^[^ ^72^ ^]^ Additionally, the healthy MO that had been cultured together with 3×SNCA HBO presented dysregulations in synaptic pathways. As a protein present in the presynaptic terminals, it has been suggested that α‐Syn should play a role in synaptic function and neurotransmitter release.^[^ ^73^ ^]^ Consequently, pathological α‐Syn has been associated with synaptic dysfunction,^[^ ^74^ ^]^ via mechanisms that can involve the mitochondria, oxidative stress induction, protein degradation systems impairment, and nuclear changes.^[^ ^75^ ^]^ We were able to confirm in the model an increase in presynaptic protein Neurexin‐1, as well as changes in the synaptic connectivity, observed as a shift in the directionality of the signals within the two assembloid compartments. The MEA experiment provides valuable insights into signal propagation within the assembloid system. However, we acknowledge that the electrode placement in the tunnels primarily captures axonal signal transmission rather than direct evidence of synaptic connections between HBO and MO. The observed temporal delays between electrodes likely reflect action potential propagation along axons of either MO or HBO neurons, rather than synaptic transmission between the two populations. While these data demonstrate physical connectivity and functional signal transmission within the assembloid, it does not conclusively prove the existence of synaptic connections between HBO and MO. Nevertheless, these synaptic connections are actually proven by the rabies virus tracing experiment. Yet we consider that the directionality of the signals could be relevant in the context of α‐Syn transport between the different brain regions, as it likely involves a combination of both afferent and efferent pathways. Our findings indicate that an early elevation of α‐Syn in the healthy midbrain can lead to a start of the pathology there, and primarily affect the synapses, even before observing dopaminergic neurodegeneration.

Altogether, the assembloid model is able to recapitulate in vitro early stages of α‐Syn pathology and its transfer, following Braak's hypothesis for PD. In the present study, we used a patient *SNCA* triplication model as the most resembling to an overexpression of α‐Syn. Yet it is known that not all patients with synucleinopathies, including PD, follow the dual‐hit entry point of the pathology or Braak's staging system. Therefore, new trends following re‐analysis of post‐mortem samples^[^ ^76^ ^]^ propose a body‐first versus brain‐first model, in which α‐Syn pathology has a single entry point, from which pathology can disperse: from the peripheral enteric nervous system to the brain or from the central nervous system to the lower brainstem and peripheral autonomic nervous system.^[^ ^77^ ^]^ Models like this, containing several components of the body's nervous system, could bring insights into understanding further aspects of α‐Syn pathology and its spread, and they could be used to identify therapeutic strategies to halt or prevent it. In this manuscript, we also explored the possibility of integrating the assembloid model into on‐chip approaches for a specific application, being able to better isolate communication between the organoids composing the assembloid. In this line, further model improvements that are already being explored, such as assembloid‐on‐a‐chip approaches^[^ ^78^ ^]^ could become very relevant in the context of PD and α‐Syn research.

## 4 Experimental Section

### Experimental Design

In this study, assembloids were employed as a tool to investigate spread of disease pathology between two region‐specific organoids. First, a novel protocol for the generation of hindbrain organoids was presented which, together with a previously published midbrain organoid protocol, were the two methods used to create the assembloids. Characterization of the organoid entailed snRNAseq, western blot, dot blot, and immunofluorescence. Retrograde viral tracing and MEA analysis were used to show functional aspects of the connectivity within the assembloids. Finally, the assembloid model was used to study α‐Syn pathology.

### hiPSC, Neuroepithelial Stem Cells, and Organoid Culture

Detailed information concerning the hiPSC lines used in this study is given in Table S1 (Supporting Information). Methodology on iPSC culturing and extensive characterization of the lines could be found in Muwanigwa et al.,^[^ ^28^ ^]^ as well as the certificate of analysis from the NIGMS Human Genetic Cell Repository for the hiPSC line GM25256*I.

For the generation of HBO, 9000 iPSCs were seeded in each well of an ultralow attachment BIOFLOAT 96 U‐bottomed well plate (faCellitate, cat no. F202003) and cultured in embryoid body (EB) medium. EB medium consisted of Dulbecco's modified eagle medium (DMEM)/F12 (Thermo Scientific, cat no. 21331‐046) containing 20% knock‐out serum replacement (KOSR, Thermo Scientific, cat no. 10828028), 3% fetal bovine serum (FBS, BWR, cat no. HYCLSH30070.03), 1% MEM nonessential amino acids (Life Technologies, cat no. 11140‐050), 1% glutamax (Thermo, cat no. 35050061), 1% Penicillin‐Streptomycin (P/S), and 0.7% 2‐mercaptoethanol (Thermo, cat no. 31350‐010). Media were filtered using a vacuum‐driven 0.2 µm Steriflip filter unit (Millipore, cat no. SCGP00525). Before use, medium was supplemented with a final concentration of 4 ng µL^−1^ basic fibroblast growth factor (bFGF) (PeproTech, cat no. 100–18B) and 50 µm Y‐27632. The next day, colony formation was assessed under the microscope. If colonies were formed, the medium was switched to neural patterning medium (NPM). NPM consisted of a 1:1 volume ratio of DMEM/F12 and neurobasal (Thermo, cat no. 21103‐049), 0.5% N_2_ supplement (Thermo, cat no. 17502001), 1% B27 supplement without vitamin A (Life Technologies, cat no. 12587001), 1% glutamax, and 1% P/S. On days 1–4, NPM medium was additionally supplemented with 10 µm final concentration of SB431542 (Abcam, cat no. ab120163), 100 ng mL^−1^ Noggin (Stem Cell Technologies, cat no. 78060) and 4 µm CHIR99021 (Axon, cat no. CT 99021), and replaced every other day. On days 4–8, NPM medium was freshly supplemented with the same concentrations of SB431542, Noggin, and CHIR99021, together with 0.5 µm PMA (Enzo, cat no. ALX‐420‐045‐M005), 500 nm retinoic acid (Sigma, cat no. R2625) and 0.5 µm SAG (Merck cat no. 566660‐1MG). On day 8, each EB was embedded in 25 µL Geltrex (Invitrogen, cat no. A1413302) and transferred to 24‐well ultralow attachment plates (CELLTREAT, cat no. 229524) and kept under differentiation media (DM) at 37 °C, 5% CO_2_ under static conditions for the first 4 days. DM composition was the same as NPM medium, only supplemented freshly with 10 ng mL^−1^ GDNF (PeproTech, cat no. 450‐10‐1 mg), 10 ng mL^−1^ BDNF (PeproTech, cat no. 450‐02 –1 mg), 500 µm cAMP (cyclic adenosine monophosphate) (Biosynth, cat no. ND07996), 200 µm ascorbic acid (AA, Sigma, cat no. A4544‐100G), 0.5 µm SAG and 2.5 µm DAPT (R&D Systems, cat no. 2634/50). From day 12 on, DM was supplemented with GDNF, BDNF, cAMP, AA, and 1 ng mL^−1^ TGFβ3 (PeproTech, cat no. 100‐36E) and HBO were placed under shaking conditions (80 rpm). Medium was then replaced every 3–4 days for up to 60 days.

For the generation of MO, neuroepithelial stem cells (NESC) were used. NESC derivation and quality assessment is detailed in Muwanigwa et al.^[^ ^28^ ^]^ MO were generated following the protocol from Zagare et al.^[^ ^33^, ^34^ ^]^ MO were embedded in 25 µL Geltrex and cultured in 24 low attachment well in an atmosphere controlled incubator and under dynamic conditions (80 rpm) for up to 60 days.

### Assembloid Generation

To generate midbrain–hindbrain (MO–HBO) assembloids, MO and HBO were generated separately and, after 30 days of differentiation, they were placed in 1.5 mL microcentrifuge tubes, as in Andersen et al.,^[^ ^35^ ^]^ to form assembloids. The final culture medium for the assembloids was DM supplemented with BDNF, GDNF, cAMP, AA, and TGFβ3, at the same concentrations a before. Organoids were kept static in microcentrifuge tubes and media were carefully changed every 3–4 days thereafter. Assembly was maintained for 30 more days, bringing the total age of the cultures to 60 days of differentiation. Afterward, assembloids were either assessed as a unit, or separated again into MO and HBO for independent analysis. Separation of the assembloids was performed visually by placing them in a cell culture Petri dish and separating the two region‐specific organoids with a scalpel (as detailed in Figure 5A). MO can be distinguished from HBO mostly by size and morphology. MO consistently develop a flat disk‐like shape after the embedding, with the round core in the centre or on one side and visible matrix surrounding it. Moreover, they remain smaller. On the contrary, the core of the HBO tends to be irregular in shape and it grows into the matrix in such a way that it eventually becomes impossible to see the surrounding matrix. Overall, HBO grow bigger (Figure S6A, Supporting Information).

### Retrograde Monosynaptic Tracing

Sequential viral labeling of the region‐specific organoids was performed to assess the connectivity through active synapses between the hindbrain and the midbrain neurons in the assembloid model. To achieve that, MO at day 20 of differentiation were placed in a 1.5 mL microcentrifuge tube and transduced by adding culture medium containing concentrated replication‐deficient LV–GP–TVA–GFP lentiviral particles. High titter preparations of lentiviral particles were produced, as previously described,^[^ ^36^ ^]^ using he construct pBOB–synP‐HTB (gift from Edward Callaway and Liqun Luo (Addgene plasmid # 30195; http://n2t.net/addgene:30195; RRID:Addgene_30195)).^[^ ^37^ ^]^ By initially labeling the MO with GFP, a population of starter cells that express both GFP and the rabies virus receptor (TVA) was created. This should ensure that the subsequent rabies virus infection will specifically initiate from these cells.

After 7 days, the medium containing the lentiviral vector was discarded and the organoids were washed twice with fresh medium, to prevent carry‐over of viral particles to the HBO. Then, a hindbrain organoid was placed into every 1.5 mL microcentrifuge tube containing every LV‐transduced MO to allow the assembly of the two organoids. Then, assembloids were transduced with the concentrated RBV–ΔG–EnvA–RFP rabies viral particles, which were achieved following an established protocol.^[^ ^38^ ^]^ The pseudotyped rabies virus should only be able to enter the cells which express the TVA receptor and spread retrogradely across functional synapses. This means it travels from the postsynaptic neuron (in the MO) to the presynaptic neuron (in the HBO) and that neurons in the hindbrain that form functional synapses onto the initially infected midbrain neurons will be labeled with RFP. Single viral infection control experiments were performed in parallel to confirm the specificity of the signal. About a week later, media containing viral particles were changed and the assembloids were cultured for up to 30 additional days, following the regular media exchanges. After this time, assembloids were fixed with 4% paraformaldehyde (PFA) and processed for immunocytochemistry (detailed procedure later on). Assembloids were sliced into 70 µm thick sections and only nuclei were stained in addition with Hoechst 33342 (Invitrogen, cat no. 62249). Sections were mounted and imaged under a confocal laser scanning microscope (Zeiss LSM710) for the observation of the RFP and GFP signals from the viral infections.

### Single‐Nuclei RNA Sequencing

For preprocessing of samples and characterization of the HBO, healthy (Ctrl_2) HBO (line 232) produced from three independent derivations were pooled (three HBO per batch) into 1.5 or 2 mL microcentrifuge tubes, snap‐frozen, and stored at −80 °C. Healthy MO from the same line were assembled together either with healthy HBO or with 3×SNCA (line 336, carrying *SNCA* triplication) HBO, as previously described. After 30 days of co‐culture/assembly, healthy MO from both conditions were separated from the HBO, to evaluate the influence on a healthy midbrain of either a healthy or a PD hindbrain. As before, three independent organoid derivations and assembloid generations were produced to make a pool of three biological replicates. Because MO are generally smaller than HBO, 9–12 MO were pooled per replicate to get enough material for analysis. To compare these samples to the PD midbrain, 3×SNCA MO were also generated from three independent organoid derivations, snap‐frozen and kept at −80 °C, until the time of processing. Single MO and HBO samples were collected at day 60 of differentiation to be consistent with the age of the MO after assembly.

Samples from the three batches were pooled for nuclei extraction and sorting. Tissues were lysed by adding 1 mL chilled lysis buffer (10 mm Tris‐HCl, 10 mm NaCl, 3 mm MgCl_2_, 0.1% Nonidet P40, 1% bovine serum albumin (BSA), and 0.2 U µL^−1^ of RNase inhibitor in nuclease‐free water). Organoids were then incubated on ice for 15–30 min while being physically disrupted by pipetting up and down gently with P1000 or P200 pipette tips a few times during the incubation. Depending on the sample size, incubation times were prolonged until no tissue remnants were visible. Then, the suspension was filtered using a 30 µm MACS SmartStrainer to remove cell debris and large clumps and nuclei were pelleted by 5 min centrifugation at 500 rcf and at 4 °C. Without disrupting the nuclei pellet, the supernatant was removed and pellets were washed in 0.7 mL nuclei wash and resuspension buffer (1× phosphate‐buffered saline (PBS), 1% BSA, and 0.2 U µL^−1^ RNase inhibitor), filtered, and the procedure was repeated. The second time, nuclei pellets were incubated for 5 min in the DAPI solution (5 µL DAPI (300 µm) in 1000 µL of 1× PBS) prior to FACS sorting. Within this incubation time, a fraction of the nuclei suspension was counted under the microscope in a Neubauer chamber using Trypan Blue, in order to evaluate the presence and the quality of the extracted nuclei. In the subsequent FACS sorting, single DAPI‐positive nuclei were selected using a calibrated FACS ARIA III Cell Sorter (BD Biosciences) and applying size and granularity filters to minimize the amount of cell debris in the suspension, using 4‐way purity sort with 32 purity mask. Nuclei were sorted with an event rate between 200 and 1000 events s^−1^. The sorted nuclei were centrifuged and pelleted as before, and nuclei were inspected under the microscope and manually counted on the Neubauer chamber. Based on the number of intact nuclei obtained, the amount of nuclei suspension buffer was adjusted to yield a single nuclei solution of ≈1000 nuclei µL^−1^ and proceeded immediately with 10× chromium.

For library preparation and sequencing, after nuclei extraction an individual lane per sample of Chromium Next GEM Single Cell 3′ Kit v3.1 chemistry was run on a chromium controller device (10× Genomics). Libraries were generated following manufacturer's recommendations. Final library concentration was measured on a Qubit device using the dsDNA HS chemistry (ThermoFisher) and library size distribution was determined using an Agilent High Sensitivity DNA Kit on a 2100 Bioanalyzer Instrument. Libraries were equimolarly pooled and clustered at 650 pm on a P3 Flowcell and subsequently sequenced R1 28 cycles, I1 10 cycles, I2 10 cycles, R2 90 cycles on a NextSeq2000 Instrument (Illumina). Data were demultiplexed and converted into fastq files using bcl2fastq2 v2.20 and subsequently single‐cell demultiplexed and converted into count matrixes using cellranger 5.0.1.

For data analysis, individual datasets of MO and HBO were integrated and analyzed using Seurat R toolkit version 4.2.0.^[^ ^39^ ^]^ on R version 4.2.2. Only nuclei having more than 500 genes with a minimum of 750 and a maximum of 25 000 unique feature counts and less than 5% of mitochondrial or ribosomal genes were retained for the analysis. Datasets were integrated considering 2500 most variable genes and 50 principal components following Seurat integration workflow.^[^ ^39^ ^]^ The Louvain algorithm modularity optimization was used with a resolution of 0.1 to identify cell clusters, and these were visualized with uniform manifold approximation and projection (UMAP).^[^ ^40^ ^]^ Cell cluster marker genes were determined using “FindAllMarkers” function of Seurat. Cellular identities of 12 cell clusters were further determined based on the marker genes using GeneAnalytics online tool,^[^ ^41^ ^]^ choosing in vitro parameter for brain cells. Validation of the identity of each cluster was done using cell type specific markers described in literature and using PanglaoDB.^[^ ^42^ ^]^ Differentially expressed genes were detected using the “FindMarkers” function of the Seurat. Significant DEG (p.adjust < 0.05 and logfc.threshold = 0.25) were selected for further enrichment analysis using MetaCore (version 2022 Clarivate). The genes were plotted from the most enriched pathways (Fold change) in GraphPad Prism 9.

### Quantitative PCR

For total RNA extraction, RNeasy Mini Kit (Qiagen, cat no. 74106), was used following the protocol provided by the manufacturer. Complementary DNA synthesis was done using the high‐capacity RNA‐to‐cDNA kit (ThermoFisher, cat no. 4387406) following manufacturer's instructions. Maxima SYBR Green qPCR Master Mix (ThermoFisher, cat no. K0221) was used together with the primers in Table S2 (Supporting Information) to characterize the HBO model. Quantitative PCR was carried out in an Aria Mx Real‐Time PCR system (Aligent) and data were extracted and analyzed in the AriaMx PC software (Agilent).

### Protein Extraction and Quantification

For fractionation of soluble and insoluble α‐synuclein, collected and snap‐frozen organoids (MO or HBO) were placed on ice and lysed following the methodology outlined in Muwanigwa et al.^[^ ^28^ ^]^

Then whole protein extraction was performed. In cases where fractionation of α‐synuclein was not required, protein was extracted using radioimmunoprecipitation assay (RIPA) buffer (Abcam, ab156034) supplemented with protease and phosphatase inhibitors, as before. Tissues were also mechanically disrupted by pipetting up and down until a homogeneous solution was obtained, while incubating them on ice. For DNA disruption, lysates were sonicated for ten cycles (30 s on/30 s off) using the Bioruptor Pico (Diagenode), followed by a centrifugation at 4 °C for 30 min at 14 000 × *g*.

For protein quantification, the protein concentration for each sample was determined using the Pierce bicinchoninic acid (BCA) protein assay kit (Thermo Fisher Scientific, cat no. 23225), according to the manufacturer's instructions. In cases where protein was quantified from the culture media for normalization purposes, the Protein Quantification Assay (Macherey‐Nagel, cat no. 740967.50) was used.

### Western Blot

Samples were normalized to equal concentrations using lysis buffer and denatured in loading buffer at 95 °C for 5 min. For each western blot, up to 20 µg of protein was loaded per sample. Proteins were separated using SDS‐PAGE (sodium dodecyl sulfate–polyacrylamide gel electrophoresis) on Bolt 4–12% bis–tris Plus gels (ThermoFisher cat no. NW04127BOX) and transferred to polyvinylidene fluoride (PVDF) membranes using iBlot 2 Gel Transfer Device (ThermoFisher). Membranes were fixed in 0.4% PFA in Tris‐buffered saline (TBS) for 30 min at room temperature (RT), followed by three 5 min TBS washes. Blocking was performed for 1 h at RT in 5% BSA powder in TBS containing 0.2% Tween. Blocking was performed in 5% BSA in TBS with 0.2% Tween for 1 h at room temperature. Primary antibody incubation was carried out overnight at 4 °C in 5% BSA with 0.02% Tween. After three 10 min washes in 0.02% Tween in TBS, membranes were incubated with DyLight secondary antibodies (1:10 000 dilution; antirabbit Immunoglobulin G (H+L) 800, Cell Signaling, cat no. 5151P or antimouse Immunoglobulin G (H+L) 680, and Cell Signaling, cat no. 5470P). Membranes were imaged using an Odyssey Fc 2800 imaging system with exposure times ranging from 30 s to 2 min. Signal intensity was quantified using ImageJ (RRID:SCR_003070) and Image Studio Lite (version 5.2) software. Uncropped membrane images are provided in Figure S9A–E (Supporting Information).

### Dot Blot for α‐Synuclein

At every collection time point, spent media from the cultured MO and HBO were collected, snap‐frozen, and stored at −80 °C. To prepare for dot blot, media were placed on ice to thaw on ice and then spun down (300 g, 4 °C, and 5 min) to allow cell debris remaining in the media to sediment. A 96‐well dot‐blot array system (Dot Blot Minifold I, Whatman, cat no. 10447900) was employed according to manufacturer guidelines. Nitrocellulose membranes (Sigma‐Aldrich, cat no. GE10600001) were used for the capture of proteins from the supernatant, after rehydration with 300 µL of PBS per well before sample loading. 300 µL of spent media was run (with vacuum ON) per sample and per well. The membrane was then retrieved and subjected to fixation, blocking, and antibody incubations using the same protocol as described for western blotting. Images were acquired with the Odyssey Fc 2800 Imaging System and analyzed with the same software. Relative α‐synuclein amount was normalized either to the protein concentration of the media or to Ponceau S (Sigma, cat no. P7170‐1L) protein staining of the membrane.

### Immunofluorescence Staining of Organoid Sections

At the dedicated time points, whole organoids were collected and fixed in 4% PFA overnight at 4 °C. After, they were washed three times with PBS for 15 min and embedded singly in 3% low‐melting point agarose (Biozym, cat no. 840100). At least three organoids per line, per batch, and for each time point were embedded. By means of a vibrating blade microtome (Leica VT1000s, RRID:SCR_016495), 70 µm thick organoid slices were obtained. Before staining, sections were permeabilized and blocked using the same buffers and conditions outlined in Muwanigwa et al.^[^ ^28^ ^]^ Primary antibody incubations (see Table S3 in the Supporting Information) were performed in blocking buffer containing 0.1% Triton X‐100 for 48 h at 4 °C. Secondary antibodies (Table S2, Supporting Information), together with Hoechst nuclei stain at 1:1000 dilution, were incubated for 2 h and at RT. After the pertinent washes, sections were mounted on Teflon‐coated slides with 24 wells ø 4 mm (de Beer Medicals, cat no. BM‐9244) using Fluoromount‐G mounting medium (VWR, cat no. SOUT0100‐01).

### Thioflavin S Staining

ThioS staining was performed by adding 0.05% ThioS w/v in 50% ethanol/water to the organoid sections for 15 min incubation at RT and protected from the light. This step was done after sections were already incubated with secondary antibodies and subsequently washed. Upon incubation, sections were washed twice with 50% ethanol in water for 20 min each and then washed once with 80% ethanol in water for another 20 min. Then, ethanol was removed and sections were washed with and mounted as explained in the anterior section. Visualization was performed with confocal microscopes (see the following section), and signal co‐localization between α‐synuclein species and ThioS was assessed.^[^ ^43^ ^]^

### Fontana–Masson Staining

To verify that the dark pigmentation observed in some organoids corresponded to neuromelanin, a Fontana‐Masson qualitative staining (Sigma, cat no. HT200) was performed. Organoids containing visible pigment were selected, sectioned into 30 µm sections using a Leica cryostat (CM1850UV), and stained observing the manufacturer's recommendations.

### Image Acquisition

For high‐content image acquisition, images of mounted samples of every staining combination were acquired using a Yokogawa CV8000 high content screening microscope (RRID:SCR_023270). In order to select the wells where there were organoids, a pre‐scan of the slide was done using a 4× objective and the 405 channel, which was normally where the nuclei were labeled, and it allowed for drawing a mask, based on this marker, around the organoid. This organoid mask is able to distinguish every section, and it is used to calculate all the fields that will be acquired in all the corresponding wavelengths with a 20× objective. As basis, for all the stainings performed in this manuscript, at least one section from three organoids of each condition and from at least three batches were used. More information about the actual number is added to the figure legends of the corresponding experiments. Qualitative images were acquired using a confocal laser scanning microscope (Zeiss LSM 710, RRID:SCR_018063) with either a 20×, 40×, or a 60× objective.

### Image Analysis

Images obtained from the Yokogawa microscope were processed and analyzed in Matlab (2021a, Mathworks, RRID:SCR_001622) using a previously described image analysis pipeline^[^ ^44^ ^]^ and customized scripts. Briefly, the custom image‐analysis algorithm was first stitched together overlapping sections of images to create a larger, complete mosaic picture. On subsequent steps, the images were smoothed and combined from different color channels, small objects, and sparse structures were removed, and this was applied to all layers of the 3D images. For each channel, corresponding to a stained marker, images were processed to enhance the contrast between the marker and the background (segmentation). Then, in order to detect the areas positive for the marker, a rough mask outline was created based on brightness levels (pixel intensities). This mask was then refined by removing false detections and very small objects. Ultimately, the defined masks were used to perform quantifications of the area occupied by each marker in the 3D space (voxels).

### Image Processing and Presentation

For some microscopy images, minor adjustments were made to improve clarity and presentation of the figures, without altering the scientific content. In cases where the original scale bar was in a suboptimal position or cropped out during image processing, the scale bar was digitally reproduced and placed in a more visible location within the same image. The length and units of the scale bar were maintained as in the original image. All image manipulations were performed using Adobe Illustrator (28.0 version). Original, unprocessed images are available (see the Supporting Information). These adjustments did not affect the scientific interpretation of the data presented.

### Multielectrode Array on Microtunnel Devices

PDMS microchannel devices^[^ ^79^ ^]^ (see the Supporting Information) were aligned with planar electrodes of commercial 12‐well MEA plates (Axion, M768‐GL1‐30Pt200) to guide axons from organoid to organoid and to create an electrically isolated more stable cellular microenvironment.^[^ ^45^ ^]^ The MEA consists of an array of 8 × 8 electrodes, each spaced 200 µm apart (Figure S5B,C, Supporting Information). Therefore, the MD was designed with tunnels also spaced 200 µm apart. Additionally, to fit the MD into the well plate and ensure the tunnels cover the electrodes, the MD was cut. The MD contained two reservoirs, which were 2 mm diameter, providing sufficient space for placing organoids into them. The distance between the centers of the reservoirs was 3.4 mm, meaning that the closest parts of the reservoirs were 1.4 mm apart. This arrangement ensured that all 8 × 8 electrodes were beneath the tunnels. Furthermore, four additional punches were made to expose the reference electrodes in the four corners of the MEA. During the manual assembly of the MDs on top of the electrode arrays (Figure 4C), a small droplet of ethanol facilitated the alignment procedure. The assembly was left at room temperature for 30 min to allow the ethanol to dry, ensuring a leak‐free bond between the MD and MEA. The tunnels were perfectly aligned with the electrodes, making it possible for neuronal extensions to extend over the electrodes and record their electrical activities.

Prior to introducing the organoids, MEA plates were coated with poly‐d‐lysine (0.1 mg mL^−1^, Sigma‐Aldrich, P7886) in sterile PBS (Thermo Fisher Scientific, 14190250) overnight at 37 °C, followed by an 1 h laminin (1 mg mL^−1^, Sigma‐Aldrich, L2020) incubation in PBS. After that, laminin coating was removed and the plates were rinsed twice with PBS. MO and HBO were generated as outlined before, with the difference that, instead of being embedded at day 8, differentiation was started in the 96 ULA plate and organoids for 20 days and then placed on precoated microtunnel devices, together with a few microliters of Geltrex. Cultures were maintained under differentiation conditions for up to 52 days, performing recordings once or twice a week. Therefore, date of measurements was expressed as DIV, after the initial 20 days of differentiation. The plate was always kept in an incubator (37 °C, 5% CO_2_) under static conditions. MO were always seeded in the left compartment, while HBO were positioned on the right compartment (Figure S5D, Supporting Information). MO were always control organoids (Ctrl_2 cell line) and HBO were either control (Ctrl_2 cell line) or PD (3×SNCA line). Each assembloid was placed in the centre of the well on the electrodes and after the medium was carefully aspirated, it was left for 2–3 min to dry. Electrophysiological activity at the different time point was recorded using the Axion Maestro Multiwell 768‐channel MEA system (Axion Biosystems) and the Axis software (Axon Biosystems, RRID:SCR_016308).

For the general analysis of the data, compiled neural statistics were exported from the Axis software (Version 2.1.1.16) and the data were plotted using GraphPad Prism (version 9). Specifically for the directional analysis, the information contained in the spike lists generated by the Axis software was used to infer signal directionality. Briefly, the spike lists contained the action potential events, recorded at a frequency of 12.5 kHz, and the time they occurred, for each electrode. In every microchannel between neuronal populations, there were up to eight electrodes situated periodically along the channel at intervals of 200 µm. The event‐time lists generated were loaded into MATLAB (2021a, Mathworks, RRID:SCR_001622) for further analysis.

Initially, propagation velocity was measured by cross‐correlating the discrete time event time signal for all electrodes within a single channel. A distribution in propagation velocities was observed, and this was assumed to be related to a distribution in axonal lengths between electrodes. Signals were not analyzed from individual axons but the activity of all neurites that extended through the microchannel was measured. The peak lags in the cross‐correlation analysis corresponded to a propagation velocity of 1.5–2 m s^−1^. Several steps were taken to ensure that only action potentials were reported. The signals recorded on the microelectrodes were filtered using standard techniques to detect spikes: high‐pass filter (Butterworth 200 Hz), low‐pass filter (Butterworth 3 kHz), and the spike detector required a signal rise of 6 standard deviations of the background noise.

To determine the directionality of the action potentials, two electrodes were required to be observed. A window was determined based on the cross‐correlation lag previously observed. The window is the expected timeframe to observe a single event travelling from one electrode to the other. This window was used to filter the event times from each electrode and remove events from an electrode that did not have a corresponding event on the other electrode. This resulted in event lists that contain only directional events. From the resulting directional event lists, the number of events travelling in each direction can be counted, and the overall direction of communication between the neuronal populations can be deduced.

### Data Processing and Statistical Analysis

The datasets included in this manuscript were processed and visualized with GraphPad Prism (version 9). In terms of data preprocessing, only on some concrete experiments (e.g., western blots) the data were normalized to the controls. Evaluation of outliers was performed in GraphPad using the ROUT (robust regression and outlier removal) method (which can find any number of outliers) set at *Q* = 1%. These outlier values were removed for the posterior analysis. In the cases where statistical significance between two groups was to be assessed, the normality distribution of the data was first tested using the recommended tests for normal distribution that the software offers: D'Agostino–Pearson omnibus normality test, Anderson–Darling test, Shapiro–Wilk normality test, and Kolmogorov–Smirnov normality test. If the data were found not normally distributed in any of these tests, a non‐parametric test was applied. Only when the data were considered to have a Gaussian distribution in these methods, a parametric test was performed, not assuming equal standard deviations. When more than two groups were compared, significance was tested using one‐way analysis of variance (ANOVA) with multiple comparison analysis. Statistically significant results were indicated when *p*‐values were *<0.05, **<0.01, ***<0.001, and ****<0.0001, respectively. Some exact *p*‐values were also shown. When data were found not significant, it was not specifically stated in the figures or it is expressed as “ns,” not significant. More information on the number (*N*) of samples, replicates, and batches is added to the Figure legends. In general, all data (except for the multielectrode array experiment) represent at least three different and independent organoid derivations. Data were presented as bar graphs showing the mean ± standard deviation (SD) or using box plots or violin plots to show the full distribution of the data. On occasion, individual data points were displayed alongside means and error bars.

## Conflict of Interest

J.C.S. is the cofounder and shareholder of OrganoTherapeutics SARL. All other authors declare they have no competing interests.

## Author Contributions

G.G.‐G. designed and executed experiments, analyzed, and interpreted data, prepared figures, and wrote the original manuscript. D.F. executed experiments, analyzed, and interpreted data. D.V. executed experiments, analyzed, and interpreted data. A.Z. and K.B. performed snRNAseq analysis. P.M.A.A. supervised the high‐content imaging workflow and contributed to script development for image analysis. G.R. contributed to the analysis of microelectrode array data. R.S.‐K. contributed to the generation of microtunnel devices and the MEA experiments. K.H. and N.K. contributed experimentally to the snRNAseq. F.P. generated the RBV and LV viruses for the rabies monosynaptic tracing experiment. R.M. and M.S. reviewed and edited the manuscript. R.L. provided scientific feedback in regular project meetings, reviewed, and edited the manuscript. J.C.S. conceived and supervised the project and edited the manuscript.

## Supporting information



Supporting Information

## Data Availability

All original and processed data related to his study are available under DOI: https://doi.org/10.17881/w3kx‐xn95. Matlab and R scripts for data analysis are available on GitHub at: https://github.com/LCSB‐DVB. All data are available in the main text or the supplementary materials.

## References

[1] H. Braak , K. Del Tredici , U. Rüb , R. A. de Vos , E. N Jansen Steur , E. Braak , Neurobiol. Aging 2003, 24, 197. 12498954 10.1016/s0197-4580(02)00065-9

[2] K. Seidel , J. Mahlke , S. Siswanto , R. Krüger , H. Heinsen , G. Auburger , M. Bouzrou , L. T. Grinberg , H. Wicht , H. W. Korf , W. den Dunnen , U. Rüb , Brain Pathol. 2014, 25, 121. 24995389 10.1111/bpa.12168 PMC4397912

[3] M. G. Spillantini , M. L. Schmidt , V. M. Lee , J. Q. Trojanowski , R. Jakes , M. Goedert , Nature 1997, 388, 839. 9278044 10.1038/42166

[4] M. H. Polymeropoulos , C. Lavedan , E. Leroy , S. E. Ide , A. Dehejia , A. Dutra , B. Pike , H. Root , J. Rubenstein , R. Boyer , E. S. Stenroos , S. Chandrasekharappa , A. Athanassiadou , T. Papapetropoulos , W. G. Johnson , A. M. Lazzarini , R. C. Duvoisin , G. Di Iorio , L. I. Golbe , R. L. Nussbaum , Science 1997, 276, 2045. 9197268 10.1126/science.276.5321.2045

[5] R. Krüger , W. Kuhn , T. Müller , D. Woitalla , M. Graeber , S. Kösel , H. Przuntek , J. T. Epplen , L. Schöls , O. Riess , Nat. Genet. 1998, 18, 106. 9462735 10.1038/ng0298-106

[6] J. J. Zarranz , J. Alegre , J. C. Gómez‐Esteban , E. Lezcano , R. Ros , I. Ampuero , L. Vidal , J. Hoenicka , O. Rodriguez , B. Atarés , V. Llorens , E. G. Tortosa , T. del Ser , D. G. Muñoz , J. G. de Yebenes , Ann. Neurol. 2004, 55, 164. 14755719 10.1002/ana.10795

[7] S. Appel‐Cresswell , C. Vilarino‐Guell , M. Encarnacion , H. Sherman , I. Yu , B. Shah , D. Weir , C. Thompson , C. Szu‐Tu , J. Trinh , J. O. Aasly , A. Rajput , A. H. Rajput , A. Jon Stoessl , M. J. Farrer , Mov. Disord. 2013, 28, 811. 23457019 10.1002/mds.25421

[8] D. Hoffman‐Zacharska , D. Koziorowski , O. A. Ross , M. Milewski , J. Poznanski , M. Jurek , Z. K. Wszolek , A. Soto‐Ortolaza , J. Slawek , P. Janik , Z. Jamrozik , A. Potulska‐Chromik , B. Jasinska‐Myga , G. Opala , A. Krygowska‐Wajs , K. Czyzewski , D. W. Dickson , J. Bal , A. Friedman , Parkinsonism Relat. Disord. 2013, 19, 1057. 23916651 10.1016/j.parkreldis.2013.07.011 PMC4055791

[9] A. P. Kiely , Y. T. Asi , E. Kara , P. Limousin , H. Ling , P. Lewis , C. Proukakis , N. Quinn , A. J. Lees , J. Hardy , T. Revesz , H. Houlden , J. L. Holton , Acta Neuropathol. 2013, 125, 753. 23404372 10.1007/s00401-013-1096-7 PMC3681325

[10] P. Pasanen , L. Myllykangas , M. Siitonen , A. Raunio , S. Kaakkola , J. Lyytinen , P. J. Tienari , M. Pöyhönen , A. Paetau , Neurobiol. Aging 2014, 35, 2180. 10.1016/j.neurobiolaging.2014.03.024 24746362

[11] A. B. Singleton , M. Farrer , J. Johnson , A. Singleton , S. Hague , J. Kachergus , M. Hulihan , T. Peuralinna , A. Dutra , R. Nussbaum , S. Lincoln , A. Crawley , M. Hanson , D. Maraganore , C. Adler , M. R. Cookson , M. Muenter , M. Baptista , D. Miller , J. Blancato , J. Hardy , K. Gwinn‐Hardy , Science 2003, 302, 841. 14593171 10.1126/science.1090278

[12] L. Fonseca‐Ornelas , J. M. S. Stricker , S. Soriano‐Cruz , B. Weykopf , U. Dettmer , C. R. Muratore , C. R. Scherzer , D. J. Selkoe , npj Parkinson's Dis. 2022, 8, 118. 36114228 10.1038/s41531-022-00380-1 PMC9481630

[13] S. Zhang , R. Zhu , B. Pan , H. Xu , M. F. Olufemi , R. J. Gathagan , Y. Li , L. Zhang , J. Zhang , W. Xiang , E. M. Kagan , X. Cao , C. Yuan , S. J. Kim , C. K. Williams , S. Magaki , H. V. Vinters , H. A. Lashuel , B. A. Garcia , E. James Petersson , J. Q. Trojanowski , V. M. Lee , C. Peng , Nat. Neurosci. 2023, 26, 213. 36690898 10.1038/s41593-022-01239-7 PMC10103650

[14] D. W. Miller , S. M. Hague , J. Clarimon , M. Baptista , K. Gwinn‐Hardy , M. R. Cookson , A. B. Singleton , Neurology 2004, 62, 1835. 15159488 10.1212/01.wnl.0000127517.33208.f4

[15] I. Wurster , C. Quadalti , M. Rossi , A. K. Hauser , C. Deuschle , C. Schulte , K. Waniek , I. Lachmann , C. la Fougere , K. Doppler , T. Gasser , B. Bender , P. Parchi , K. Brockmann , npj Parkinson's Dis. 2022, 8, 117. 36109514 10.1038/s41531-022-00379-8 PMC9476413

[16] E. Vasili , A. Dominguez‐Meijide , M. Flores‐León , M. Al‐Azzani , A. Kanellidi , R. Melki , L. Stefanis , T. F. Outeiro , Mol. Neurobiol. 2022, 59, 1273. 34984585 10.1007/s12035-021-02713-2 PMC8857012

[17] J. C. Sang , E. Hidari , G. Meisl , R. T. Ranasinghe , M. G. Spillantini , D. Klenerman , Commun. Biol. 2021, 4, 613. 34021258 10.1038/s42003-021-02126-w PMC8139990

[18] R. Pinto‐Costa , E. Harbachova , P. La Vitola , D. A. Di Monte , Neurotherapeutics 2022, 20, 83. 36512255 10.1007/s13311-022-01332-6 PMC10119350

[19] E. Angot , J. A. Steiner , C. M. Lema Tomé , P. Ekström , B. Mattsson , A. Björklund , P. Brundin , PLoS One 2012, 7, e39465. 22737239 10.1371/journal.pone.0039465 PMC3380846

[20] W. Peelaerts , L. Bousset , A. Van der Perren , A. Moskalyuk , R. Pulizzi , M. Giugliano , C. Van den Haute , R. Melki , V. Baekelandt , Nature 2015, 522, 340. 26061766 10.1038/nature14547

[21] S. Zhang , E. Eitan , T. Y. Wu , M. P. Mattson , Neurobiol. Aging 2017, 61, 52. 29035751 10.1016/j.neurobiolaging.2017.09.016 PMC5705257

[22] H. Scheiblich , C. Dansokho , D. Mercan , S. V. Schmidt , L. Bousset , L. Wischhof , F. Eikens , A. Odainic , J. Spitzer , A. Griep , S. Schwartz , D. Bano , E. Latz , R. Melki , M. T. Heneka , Cell 2021, 184, 5089. 34555357 10.1016/j.cell.2021.09.007 PMC8527836

[23] M. X. Henderson , E. J. Cornblath , A. Darwich , B. Zhang , H. Brown , R. J. Gathagan , R. M. Sandler , D. S. Bassett , J. Q. Trojanowski , V. M. Y. Lee , Nat. Neurosci. 2019, 22, 1248. 31346295 10.1038/s41593-019-0457-5 PMC6662627

[24] S. Kim , S. H. Kwon , T. I. Kam , N. Panicker , S. S. Karuppagounder , S. Lee , J. H. Lee , W. R. Kim , M. Kook , C. A. Foss , C. Shen , H. Lee , S. Kulkarni , P. J. Pasricha , G. Lee , M. G. Pomper , V. L. Dawson , T. M. Dawson , H. S. Ko , Neuron 2019, 103, 627. 31255487 10.1016/j.neuron.2019.05.035 PMC6706297

[25] J. Y. Vargas , C. Grudina , C. Zurzolo , Ageing Res. Rev. 2019, 50, 89. 30690184 10.1016/j.arr.2019.01.012

[26] J. P. Anderson , D. E. Walker , J. M. Goldstein , R. de Laat , K. Banducci , R. J. Caccavello , R. Barbour , J. Huang , K. Kling , M. Lee , L. Diep , P. S. Keim , X. Shen , T. Chataway , M. G. Schlossmacher , P. Seubert , D. Schenk , S. Sinha , W. P. Gai , T. J. Chilcote , J. Biol. Chem. 2006, 281, 29739. 16847063 10.1074/jbc.M600933200

[27] N. V. Mohamed , J. Sirois , J. Ramamurthy , M. Mathur , P. Lépine , E. Deneault , G. Maussion , M. Nicouleau , C. X. Chen , N. Abdian , V. Soubannier , E. Cai , H. Nami , R. A. Thomas , D. Wen , M. Tabatabaei , L. K. Beitel , K. Singh Dolt , J. Karamchandani , J. A. Stratton , T. Kunath , E. A. Fon , T. M. Durcan , Brain Commun. 2021, 3, fcab223. 34632384 10.1093/braincomms/fcab223 PMC8495137

[28] M. N. Muwanigwa , J. Modamio‐Chamarro , P. M. A. Antony , G. Gomez‐Giro , R. Krüger , S. Bolognin , J. C. Schwamborn , Mol. Cell. Neurosci. 2024, 128, 103919. 38307302 10.1016/j.mcn.2024.103919

[29] L. R. Hernandez‐Miranda , T. Müller , C. Birchmeier , Dev. Biol. 2016, 432, 34. 27742210 10.1016/j.ydbio.2016.10.008

[30] K. Muguruma , A. Nishiyama , H. Kawakami , K. Hashimoto , Y. Sasai , Cell Rep. 2015, 10, 537. 25640179 10.1016/j.celrep.2014.12.051

[31] N. Eura , T. K. Matsui , J. Luginbühl , M. Matsubayashi , H. Nanaura , T. Shiota , K. Kinugawa , N. Iguchi , T. Kiriyama , C. Zheng , T. Kouno , Y. J. Lan , P. Kongpracha , P. Wiriyasermkul , Y. M. Sakaguchi , R. Nagata , T. Komeda , N. Morikawa , F. Kitayoshi , M. Jong , S. Kobashigawa , M. Nakanishi , M. Hasegawa , Y. Saito , T. Shiromizu , Y. Nishimura , T. Kasai , M. Takeda , H. Kobayashi , Y. Inagaki , et al., Front. Neurosci. 2020, 14, 538. 32670003 10.3389/fnins.2020.00538 PMC7332712

[32] P. Valiulahi , V. Vidyawan , L. Puspita , Y. Oh , V. B. Juwono , P. Sittipo , G. Friedlander , D. Yahalomi , J. W. Sohn , Y. K. Lee , J. K. Yoon , J. W. Shim , Stem Cell Rep. 2021, 16, 1938. 10.1016/j.stemcr.2021.06.006 PMC8365029 34242615

[33] A. Zagare , M. Gobin , A. S. Monzel , J. C. Schwamborn , STAR Protoc. 2021, 2, 100524. 34027482 10.1016/j.xpro.2021.100524 PMC8121770

[34] A. S. Monzel , L. M. Smits , K. Hemmer , S. Hachi , E. L. Moreno , T. van Wuellen , J. Jarazo , J. Walter , I. Brüggemann , I. Boussaad , E. Berger , R. M. T. Fleming , S. Bolognin , J. C. Schwamborn , Stem Cell Rep. 2017, 8, 1144. 10.1016/j.stemcr.2017.03.010 PMC5425618 28416282

[35] J. Andersen , O. Revah , Y. Miura , N. Thom , N. D. Amin , K. W. Kelley , M. Singh , X. Chen , M. V. Thete , E. M. Walczak , H. Vogel , H. C. Fan , S. P. Paşca , Cell 2020, 183, 1913. 33333020 10.1016/j.cell.2020.11.017 PMC8711252

[36] R. H. Kutner , X. Y. Zhang , J. Reiser , Nat. Protoc. 2009, 4, 495. 19300443 10.1038/nprot.2009.22

[37] K. Miyamichi , F. Amat , F. Moussavi , C. Wang , I. Wickersham , N. R. Wall , H. Taniguchi , B. Tasic , Z. J. Huang , Z. He , E. M. Callaway , M. A. Horowitz , L. Luo , Nature 2011, 472, 191. 21179085 10.1038/nature09714 PMC3073090

[38] F. Osakada , E. M. Callaway , Nat. Protoc. 2013, 8, 1583. 23887178 10.1038/nprot.2013.094 PMC4028848

[39] T. Stuart , A. Butler , P. Hoffman , C. Hafemeister , E. Papalexi , W. M. Mauck 3rd , Y. Hao , M. Stoeckius , P. Smibert , R. Satija , Cell 2019, 177, 1888. 31178118 10.1016/j.cell.2019.05.031 PMC6687398

[40] E. Becht , L. McInnes , J. Healy , C. A. Dutertre , I. W. H. Kwok , L. G. Ng , F. Ginhoux , E. W. Newell , Nat. Biotechnol. 2018, 37, 38. 10.1038/nbt.4314 30531897

[41] S. Ben‐Ari Fuchs , I. Lieder , G. Stelzer , Y. Mazor , E. Buzhor , S. Kaplan , Y. Bogoch , I. Plaschkes , A. Shitrit , N. Rappaport , A. Kohn , R. Edgar , L. Shenhav , M. Safran , D. Lancet , Y. Guan‐Golan , D. Warshawsky , R. Shtrichman , OMICS 2016, 20, 139. 26983021 10.1089/omi.2015.0168 PMC4799705

[42] O. Franzén , L. M. Gan , J. L. M. Björkegren , Database 2019, 2019, baz046. 30951143 10.1093/database/baz046 PMC6450036

[43] I. Stojkovska , W. Y. Wani , F. Zunke , N. R. Belur , E. A. Pavlenko , N. Mwenda , K. Sharma , L. Francelle , J. R. Mazzulli , Neuron 2021, 110, 436. 34793693 10.1016/j.neuron.2021.10.032 PMC8815333

[44] S. Bolognin , M. Fossépré , X. Qing , J. Jarazo , J. Scancar , E. L. Moreno , S. L. Nickels , K. Wasner , N. Ouzren , J. Walter , A. Grünewald , E. Glaab , L. Salamanca , R. M. T. Fleming , P. M. A. Antony , J. C. Schwamborn , Adv. Sci. 2019, 6, 1800927. 10.1002/advs.201800927 PMC6325628 30643711

[45] R. Habibey , S. Latifi , H. Mousavi , M. Pesce , E. Arab‐Tehrany , A. Blau , Sci. Rep. 2017, 7, 8558. 28819130 10.1038/s41598-017-09033-3 PMC5561146

[46] P. Rifes , M. Isaksson , G. S. Rathore , P. Aldrin‐Kirk , O. K. Møller , G. Barzaghi , J. Lee , K. L. Egerod , D. M. Rausch , M. Parmar , T. H. Pers , T. Laurell , A. Kirkeby , Nat. Biotechnol. 2020, 38, 1265. 32451506 10.1038/s41587-020-0525-0 PMC7616963

[47] K. Imaizumi , T. Sone , K. Ibata , K. Fujimori , M. Yuzaki , W. Akamatsu , H. Okano , Stem Cell Rep. 2015, 5, 1010. 10.1016/j.stemcr.2015.10.005 PMC4682123 26549851

[48] S. M. Chambers , C. A. Fasano , E. P. Papapetrou , M. Tomishima , M. Sadelain , L. Studer , Nat. Biotechnol. 2009, 27, 275. 19252484 10.1038/nbt.1529 PMC2756723

[49] C. Kiecker , A. Lumsden , Annu. Rev. Neurosci. 2012, 35, 347. 22462542 10.1146/annurev-neuro-062111-150543

[50] E. S. Lippmann , C. E. Williams , D. A. Ruhl , M. C. Estevez‐Silva , E. R. Chapman , J. J. Coon , R. S. Ashton , Stem Cell Rep. 2015, 4, 632. 10.1016/j.stemcr.2015.02.018 PMC4400649 25843047

[51] S. T. Waters , M. Lewandoski , Development 2006, 133, 1991. 16651541 10.1242/dev.02364

[52] R. L. Stornetta , C. J. Macon , T. M. Nguyen , M. B. Coates , P. G. Guyenet , Brain Struct. Funct. 2012, 218, 455. 22460939 10.1007/s00429-012-0408-3 PMC3459297

[53] B. A. Bari , V. Chokshi , K. Schmidt , Neural Regener. Res. 2020, 15, 1006. 10.4103/1673-5374.270297 PMC7034292 31823870

[54] K. Taylor , E. Lester , B. Hudson , S. Ritter , Physiol. Behav. 2007, 90, 744. 17289093 10.1016/j.physbeh.2006.12.014 PMC2706100

[55] L. Tricoire , T. Vitalis , Front. Neural Circuits 2012, 6, 82. 23227003 10.3389/fncir.2012.00082 PMC3514612

[56] D. D. Kline , K. N. Takacs , E. Ficker , D. L. Kunze , J. Neurophysiol. 2002, 88, 2736. 12424308 10.1152/jn.00224.2002

[57] R. L. Haining , A.‐M. C. Neuromelanin , Neural Regener. Res. 2017, 12, 372. 10.4103/1673-5374.202928 PMC5399705 28469642

[58] A. Fiorenzano , E. Sozzi , M. Birtele , J. Kajtez , J. Giacomoni , F. Nilsson , A. Bruzelius , Y. Sharma , Y. Zhang , B. Mattsson , J. Emnéus , D. R. Ottosson , P. Storm , M. Parmar , Nat. Commun. 2021, 12, 7302. 34911939 10.1038/s41467-021-27464-5 PMC8674361

[59] B. A. Hijaz , L. A. Volpicelli‐Daley , Mol. Neurodegener. 2020, 15, 19. 32143659 10.1186/s13024-020-00368-6 PMC7060612

[60] S. Grealish , A. Heuer , T. Cardoso , A. Kirkeby , M. Jönsson , J. Johansson , A. Björklund , J. Jakobsson , M. Parmar , Stem Cell Rep. 2015, 4, 975. 10.1016/j.stemcr.2015.04.011 PMC4471831 26004633

[61] G. Ugolini , J. Comp. Neurol. 1995, 356, 457. 7642806 10.1002/cne.903560312

[62] R. Etessami , K. K. Conzelmann , B. Fadai‐Ghotbi , B. Natelson , H. Tsiang , P. E. Ceccaldi , J. Gen. Virol. 2000, 81, 2147. 10950970 10.1099/0022-1317-81-9-2147

[63] T. T. Kanagasabapathi , D. Ciliberti , S. Martinoia , W. J. Wadman , M. M. Decré , Front. Neuroeng. 2011, 4, 13. 22025913 10.3389/fneng.2011.00013 PMC3198030

[64] C. Román‐Vendrell , A. T. Medeiros , J. B. Sanderson , H. Jiang , T. Bartels , J. R. Morgan , Front. Neurosci. 2021, 15, 639414. 33613189 10.3389/fnins.2021.639414 PMC7890186

[65] T. C. Südhof , Nature 2008, 455, 903. 18923512 10.1038/nature07456 PMC2673233

[66] L. M. Smits , S. Magni , K. Kinugawa , K. Grzyb , J. Luginbühl , S. Sabate‐Soler , S. Bolognin , J. W. Shin , E. Mori , A. Skupin , J. C. Schwamborn , Cell Tissue Res. 2020, 382, 463. 32737576 10.1007/s00441-020-03249-y PMC7683480

[67] I. Stojkovska , J. R. Mazzulli , STAR Protoc. 2021, 2, 100372. 33733241 10.1016/j.xpro.2021.100372 PMC7941090

[68] J. Jo , L. Yang , H. D. Tran , W. Yu , A. X. Sun , Y. Y. Chang , B. C. Jung , S. J. Lee , T. Y. Saw , B. Xiao , A. T. T. Khoo , L. P. Yaw , J. J. Xie , H. Lokman , W. Y. Ong , G. G. Y. Lim , K. L. Lim , E. K. Tan , H. H. Ng , H. S. Je , Ann. Neurol. 2021, 90, 490. 34288055 10.1002/ana.26166 PMC9543721

[69] H. Fukusumi , K. Togo , M. Sumida , M. Nakamori , S. Obika , K. Baba , T. Shofuda , D. Ito , H. Okano , H. Mochizuki , Y. Kanemura , FEBS Open Bio 2021, 11, 354. 10.1002/2211-5463.13060 PMC7876504 33301617

[70] R. Rusconi , A. Ulusoy , H. Aboutalebi , D. A. Di Monte , Aging Cell 2018, 17, e12727. 29383832 10.1111/acel.12727 PMC5847868

[71] I. C. Brás , T. F. Outeiro , Cells 2021, 10, 375.33673034

[72] N. Ramalingam , S. X. Jin , T. E. Moors , L. Fonseca‐Ornelas , K. Shimanaka , S. Lei , H. P. Cam , A. H. Watson , L. Brontesi , L. Ding , D. Y. Hacibaloglu , H. Jiang , S. J. Choi , E. Kanter , L. Liu , T. Bartels , S. Nuber , D. Sulzer , E. V. Mosharov , W. V. Chen , S. Li , D. J. Selkoe , U. Dettmer , npj Parkinson's Dis. 2023, 9, 4. 36646701 10.1038/s41531-023-00444-w PMC9842642

[73] D. L. Fortin , V. M. Nemani , S. M. Voglmaier , M. D. Anthony , T. A. Ryan , R. H. Edwards , J. Neurosci. 2005, 25, 10913. 16306404 10.1523/JNEUROSCI.2922-05.2005 PMC6725870

[74] M. G. Choi , M. J. Kim , D. G. Kim , R. Yu , Y. N. Jang , W. J. Oh , PLoS One 2018, 13, e0195339. 29608598 10.1371/journal.pone.0195339 PMC5880409

[75] K. A. Malkus , E. Tsika , H. Ischiropoulos , Mol. Neurodegener. 2009, 4, 24. 19500376 10.1186/1750-1326-4-24 PMC2701947

[76] P. Borghammer , M. K. Just , J. Horsager , C. Skjærbæk , A. Raunio , E. H. Kok , S. Savola , S. Murayama , Y. Saito , L. Myllykangas , N. Van Den Berge , npj Parkinson's Dis. 2022, 8, 166. 36450732 10.1038/s41531-022-00436-2 PMC9712280

[77] P. Borghammer , J. Horsager , K. Andersen , N. Van Den Berge , A. Raunio , S. Murayama , L. Parkkinen , L. Myllykangas , Neurobiol. Dis. 2021, 161, 105557. 34763110 10.1016/j.nbd.2021.105557

[78] Y. Zhu , X. Zhang , L. Sun , Y. Wang , Y. Zhao , Adv. Mater. 2023, 35, 2210083. 10.1002/adma.202210083 36634089

[79] A. Bastiaens , R. Sabahi‐Kaviani , R. Luttge , Front. Neurosci. 2020, 14, 666. 32670014 10.3389/fnins.2020.00666 PMC7326937

