# Supplementary material for: α‐Synuclein Pathology Spreads in a Midbrain–Hindbrain Assembloid Model
Source: Adv Sci (Weinh). 2025 Apr 17;12(20):2409040. doi: 10.1002/advs.202409040 (PMC12120764; doi:10.1002/advs.202409040)
Supplement: Supplementary file 1 — Supporting Information [file ADVS-12-2409040-s001.pdf]

ADVANCED SCIENCE
Open Access

Supporting Information

for *Adv. Sci.*, DOI 10.1002/advs.202409040

α-Synuclein Pathology Spreads in a Midbrain–Hindbrain Assembloid Model

*Gemma Gomez-Giro, Daniela Frangenberg, Daniela Vega, Alise Zagare, Kyriaki Barmpa, Paul M. A. Antony, Graham Robertson, Rahman Sabahi-Kaviani, Kristian Haendler, Nathalie Kruse, Florentia Papastefanaki, Rebecca Matsas, Malte Spielmann, Regina Luttge and Jens C. Schwamborn**

Supplementary Materials for

Alpha-synuclein pathology spreads in a midbrain-hindbrain assembloid model

Gemma Gomez-Giro *et al.*

*Corresponding author. Email: jens.schwamborn@uni.lu

This PDF file includes:

Supplementary Figures
Figs. S1 to S9
Supplementary Methods
Figs. SM1
Tables S1 to S4
References (79)

Supplementary Figures

Figure S1. Extended characterization of hindbrain organoids. (A) Representative images from Fontana-Masson staining in hindbrain organoid 70µm sections at 90 days of differentiation taken using a Nikon SMZ25 Stereomicroscope. Magnified images of regions of interest were taken at 13x magnification. Scale bars = 200 pixels. **(B)** Representative western blot for noradrenergic transporter (NET) in 30 days of differentiation HBO showing less expression in Ctrl_2 line. **(C)** Quantification of NET protein expression across 3 different derivations of HBO. Every dot represents one experiment. Data is shown as boxplots and was found not significant on a One-way ANOVA ($p=0.2998$). **(D)** Representative microscopy of glia markers GFAP and S100β in HBO sections of all the lines and at two time-points during the differentiation. Scale bars = 200µm. **(E)** Quantification of glia cell markers GFAP and S100β. Comparison was performed between lines within the same time-point and between the same line and the two different time-points, employing a One-way ANOVA with multiple comparisons (*$p<0.05$, **$p<0.01$, ***$p<0.001$, ****$p<0.0001$, ns is not significant). **(D)** Quantification of different neuronal cell type markers across lines and time-points. Comparison was performed between lines within the same time-point and between the same line and the two different time-points, employing a One-way ANOVA with multiple comparisons (*$p<0.05$, **$p<0.01$, ***$p<0.001$, ****$p<0.0001$, ns is not significant). All markers were tested, but only in those with significant groups the statistics are shown. All the other comparisons were not significant.

Figure S1.

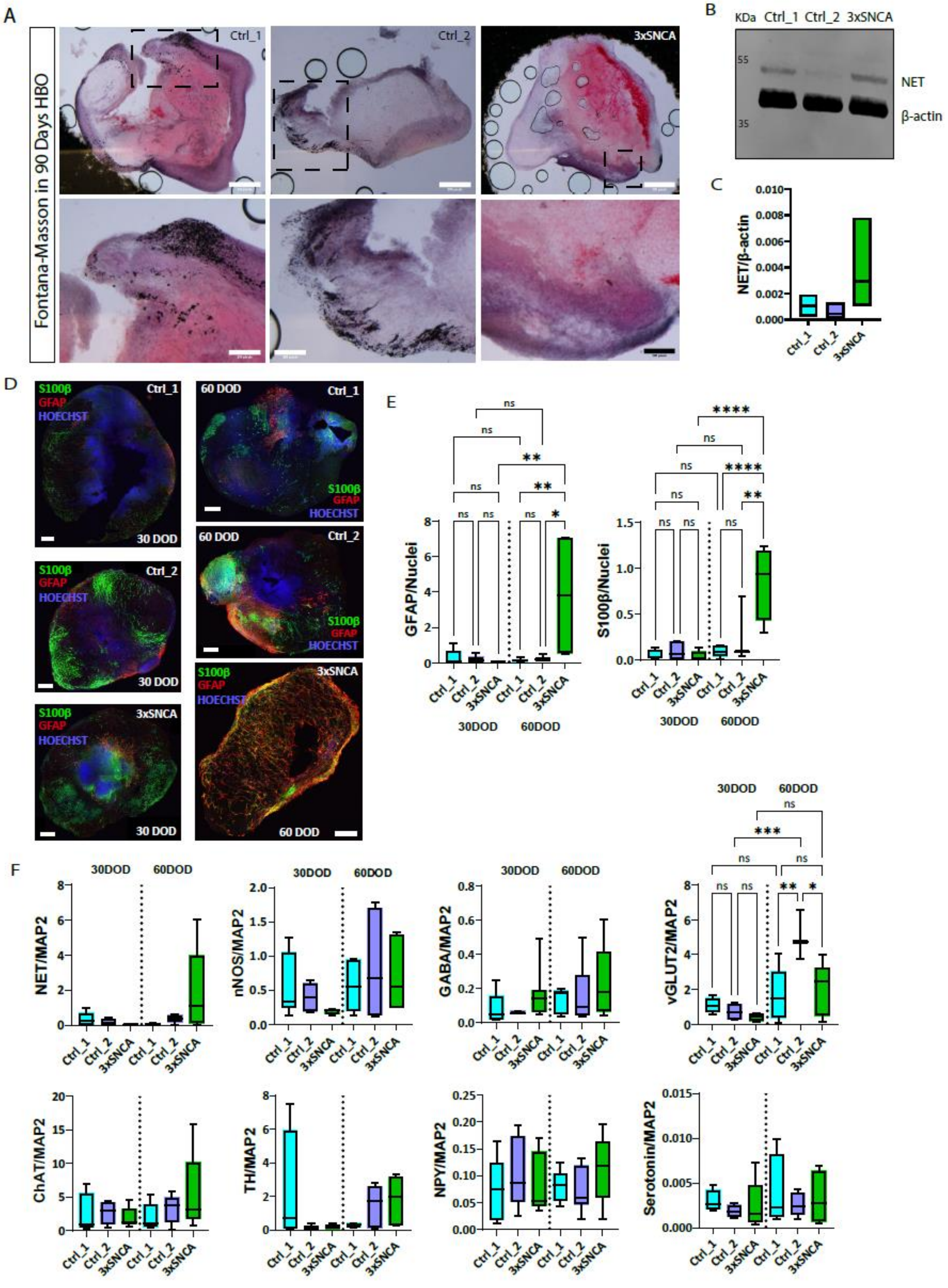
A
Fontana-Masson in 90 Days HBO
Ctrl_1
Ctrl_2
3xSNCA
B
KDa Ctrl_1 Ctrl_2 3xSNCA
55
35
NET
β-actin
C
NET/β-actin
D
S100β
GFAP
HOECHST
30 DOD
60 DOD
E
GFAP/Nuclei
S100β/Nuclei
30DOD
60DOD
ns
F
NET/MAP2
nNOS/MAP2
GABA/MAP2
vGLUT2/MAP2
ChAT/MAP2
TH/MAP2
NPY/MAP2
Serotonin/MAP2

Figure S2. Single nuclei RNA sequencing of hindbrain organoid populations. (A) Unsupervised hierarchical clustering of cell clusters based on their gene expression profile. **(B)** Dot plots showing a selection of genes that identify specifically astrocyte populations. **(C)** Dot plots showing a selection of genes that identify specifically oligodendrocyte populations.

Figure S2.

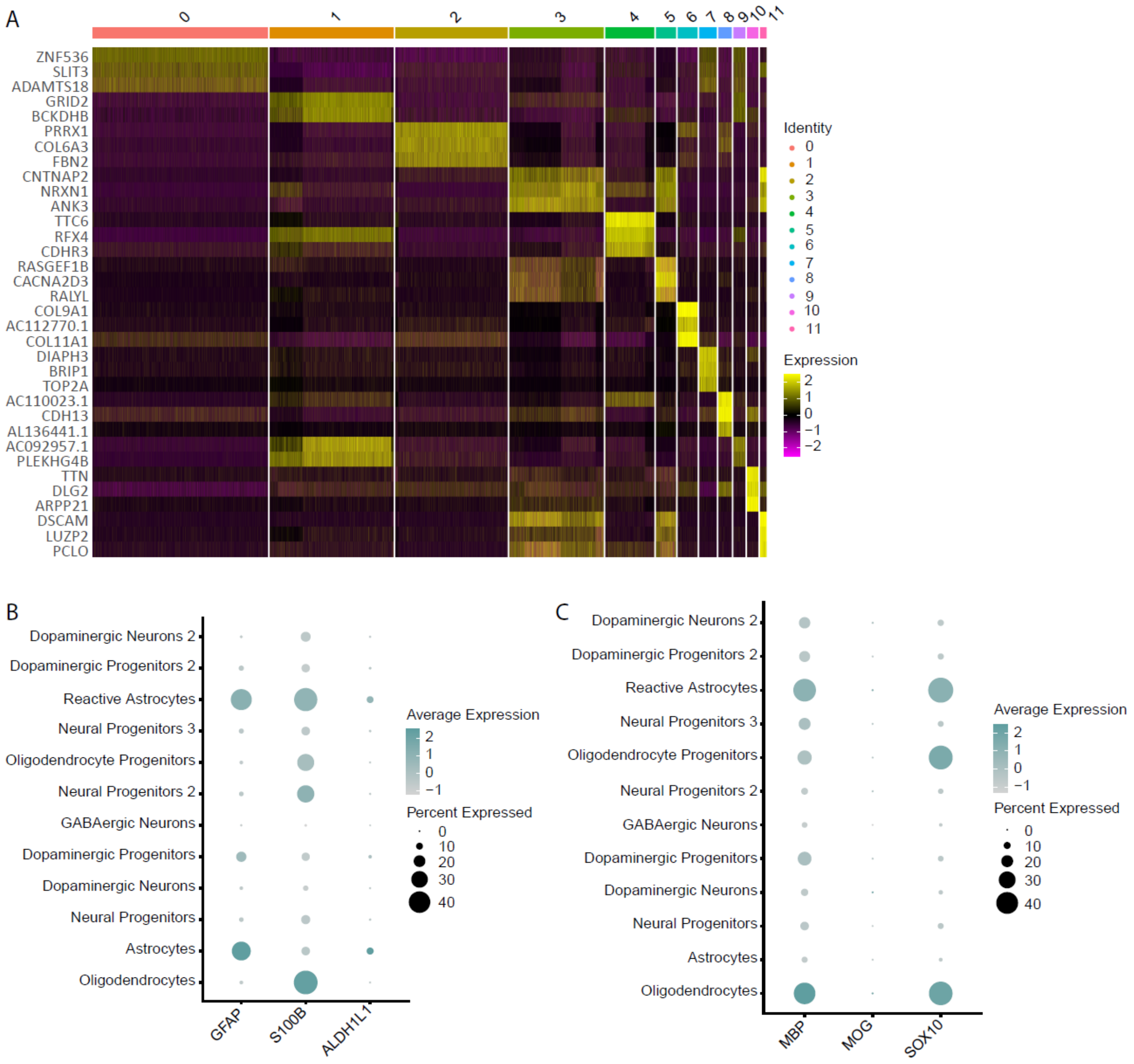
A
0 1 2 3 4 5 6 7 8 9 10 11
ZNF536
SLIT3
ADAMTS18
GRID2
BCKDHB
PRRX1
COL6A3
FBN2
CNTNAP2
NRXN1
ANK3
TTC6
RFX4
CDHR3
RASGEF1B
CACNA2D3
RALYL
COL9A1
AC112770.1
COL11A1
DIAPH3
BRIP1
TOP2A
AC110023.1
CDH13
AL136441.1
AC092957.1
PLEKHG4B
TTN
DLG2
ARPP21
DSCAM
LUZP2
PCLO
Identity
Expression
B
Dopaminergic Neurons 2
Dopaminergic Progenitors 2
Reactive Astrocytes
Neural Progenitors 3
Oligodendrocyte Progenitors
Neural Progenitors 2
GABAergic Neurons
Dopaminergic Progenitors
Dopaminergic Neurons
Neural Progenitors
Astrocytes
Oligodendrocytes
GFAP
S100B
ALDH1L1
Average Expression
Percent Expressed
C
MBP
MOG
SOX10

Figure S3. Extended single nuclei RNA sequencing data of hindbrain organoid populations. (A) Dot plots showing the expression of genes that identify mature neurons in the different cluster populations. **(B)** Dot plots showing the expression of genes that identify different neuronal populations. **(C)** Dot plots showing the expression of genes identifying dopaminergic neurons. **(D)** Comparison of expression levels of selected hindbrain genes in the HBO compared to healthy MO. For this comparison, data from MO that had been assembled with healthy HBO (H-H) was used.

Figure S3.

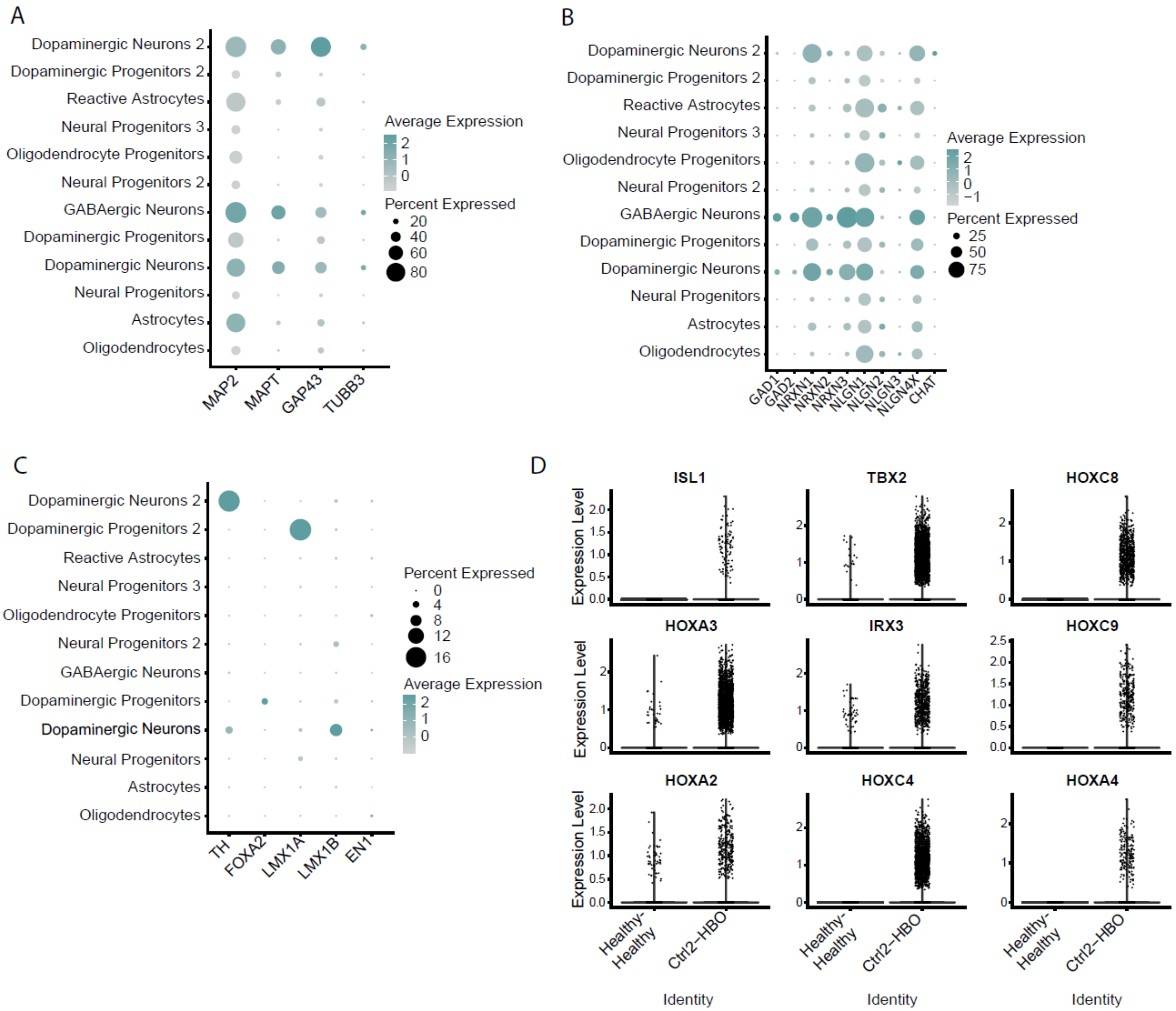

A
Dopaminergic Neurons 2
Dopaminergic Progenitors 2
Reactive Astrocytes
Neural Progenitors 3
Oligodendrocyte Progenitors
Neural Progenitors 2
GABAergic Neurons
Dopaminergic Progenitors
Dopaminergic Neurons
Neural Progenitors
Astrocytes
Oligodendrocytes
MAP2
MAPT
GAP43
TUBB3
Average Expression
2
1
0
Percent Expressed
20
40
60
80
B
GAD1
GAD2
NRXN1
NRXN2
NRXN3
NLGN1
NLGN2
NLGN3
NLGN4X
CHAT
Average Expression
2
1
0
−1
Percent Expressed
25
50
75
C
TH
FOXA2
LMX1A
LMX1B
EN1
Percent Expressed
0
4
8
12
16
Average Expression
2
1
0
D
ISL1
TBX2
HOXC8
HOXA3
IRX3
HOXC9
HOXA2
HOXC4
HOXA4
Expression Level
Healthy-Healthy
Ctrl2–HBO
Identity

Figure S4. Increased pS129 α-synuclein in 3xSNCA hindbrain organoids. (A) Microscopy images of pS129 and Thioflavin S (ThioS) signal in HBO 70µm sections at 30 days of organoid differentiation in control (CTRL) and PD (3xSNCA) conditions. Scale bar = 200µm. **(B)** Image quantification of pS129 signal intensity found in ThioS positive voxels. Control lines are pooled. HBO sections were taken from different organoids from at least 3 different organoid generations. Statistical significance by Mann-Whitney U test: ***$p < 0.001$. Squared area delimitates a zoomed in region. **(C)** Representative dot blot depicting extracellular release of α-Syn in Control and 3xSNCA HBO spent medium. SNCAKO spent medium is used as a negative control. **(D)** Dot blot quantification for the different cell lines, showing an increased α-Syn release into the medium of 3xSNCA HBO. Signal intensity of the antibody in the membrane is normalized to amount of protein in the medium. When not stated statistics are not significant. **(E)** Representative images of TH dopaminergic neurons in control and 3xSNCA 70µm HBO sections, highlighting no decrease of dopaminergic neurons in 3xSNCA HBO at 30 days of organoid maturation. Scale bar = 200µm. **(F)** Image quantification of TH signal intensity. Control lines are pooled. HBO sections were taken from different organoids from at least 3 different organoid generations. Control lines are pooled. When not stated statistics are not significant. **(G)** Representative immunoblot stained for TUJ1 and TH, specific for neurons and dopaminergic neurons respectively. **(H)** Quantifications show no decrease of the dopaminergic population in 3xSNCA HBO with respect to the controls. Control lines are pooled. When not stated statistics are not significant.

Figure S4.

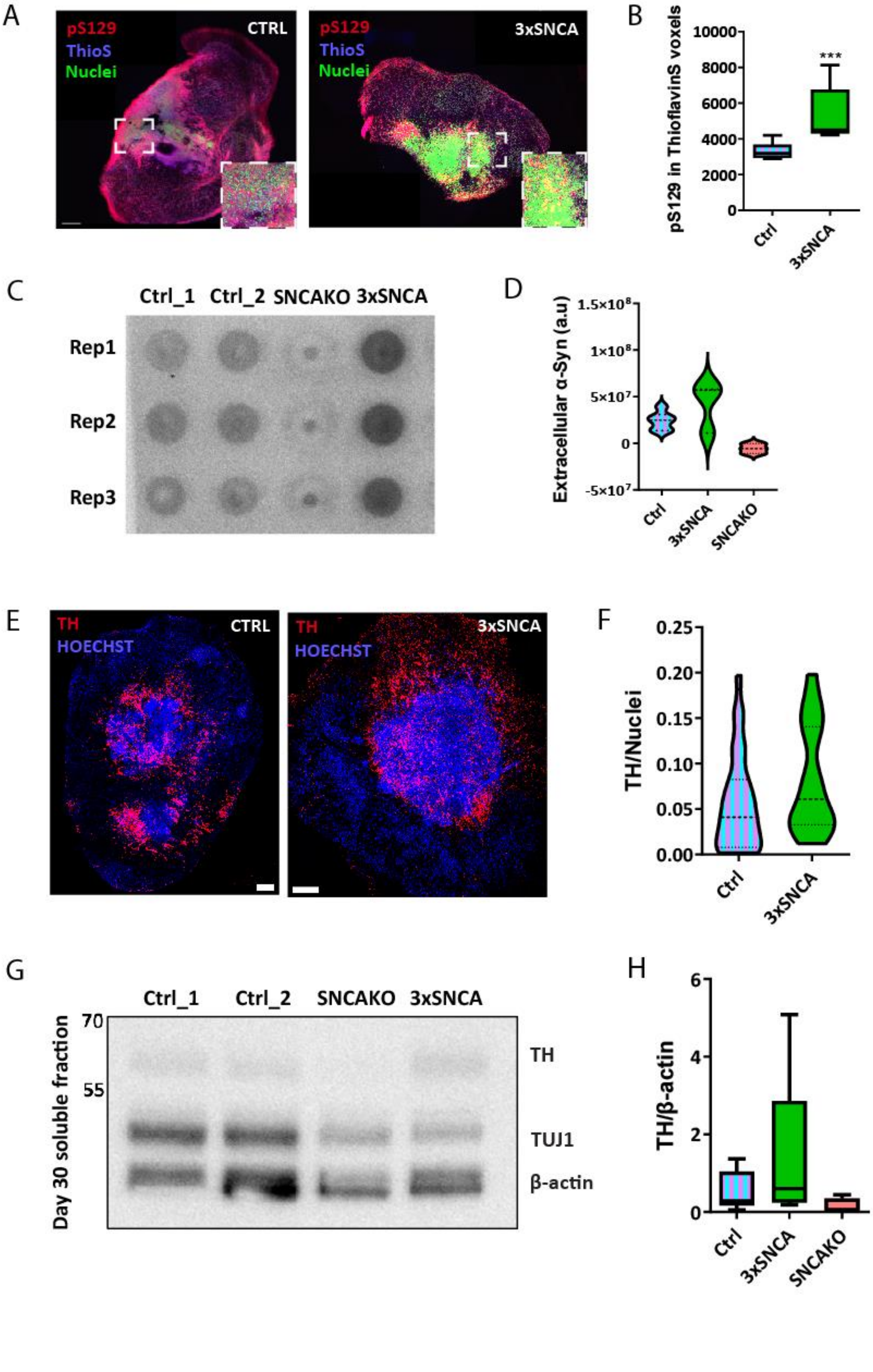
A
pS129
ThioS
Nuclei
CTRL
3xSNCA
B
pS129 in ThioflavinS voxels
10000
8000
6000
4000
2000
0
***
Ctrl
3xSNCA
C
Ctrl_1
Ctrl_2
SNCAKO
3xSNCA
Rep1
Rep2
Rep3
D
Extracellular α-Syn (a.u)
1.5×10⁸
1×10⁸
5×10⁷
0
-5×10⁷
Ctrl
3xSNCA
SNCAKO
E
TH
HOECHST
CTRL
3xSNCA
F
TH/Nuclei
0.25
0.20
0.15
0.10
0.05
0.00
Ctrl
3xSNCA
G
Ctrl_1
Ctrl_2
SNCAKO
3xSNCA
70
55
Day 30 soluble fraction
TH
TUJ1
β-actin
H
TH/β-actin
6
4
2
0
Ctrl
3xSNCA
SNCAKO

Figure S5. Extended characterization of α-Synuclein in hindbrain organoids. (A-I) Representative microscopy of the different neuronal markers and α-Synuclein in HBO at 60 days of differentiation. Zoomed regions are delimited by a dashed square. Scale bars = 200µm. **(J)** Quantification of α-Synuclein intensity in colocalization with every single neuronal cell type markers across lines and time-points. Comparison was performed between lines within the same time-point and between the same line and the two different time-points, employing a One-way ANOVA with multiple comparisons (*p<0.05, **p<0.01, ***p<0.001, ns is not significant). All markers were tested, but only in those with significant groups the statistics are shown. All the other comparisons were not significant.

Figure S5.

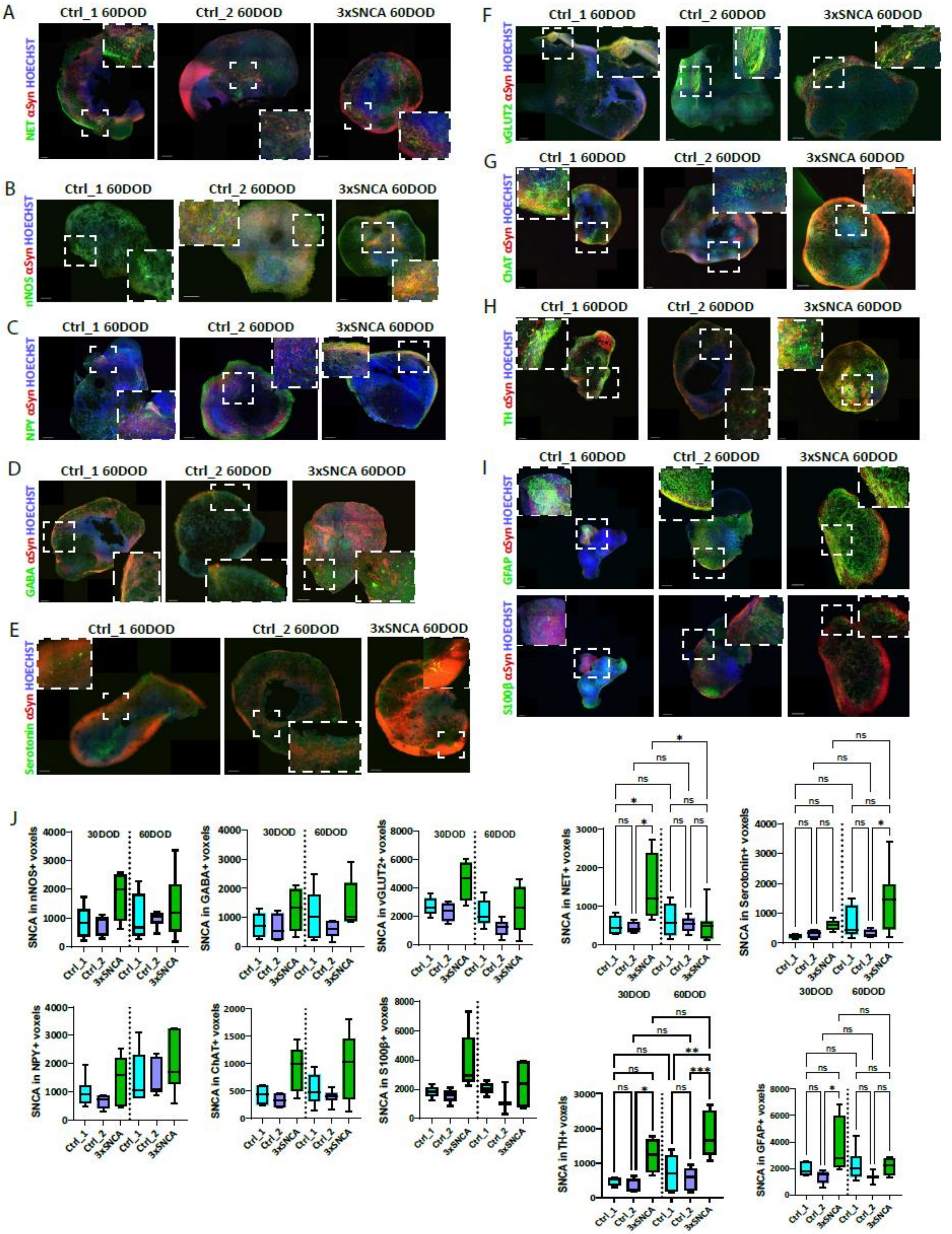
A
Ctrl_1 60DOD
Ctrl_2 60DOD
3xSNCA 60DOD
NET αSyn HOECHST
B
nNOS αSyn HOECHST
C
NPY αSyn HOECHST
D
GABA αSyn HOECHST
E
Serotonin αSyn HOECHST
F
vGLUT2 αSyn HOECHST
G
ChAT αSyn HOECHST
H
TH αSyn HOECHST
I
GFAP αSyn HOECHST
S100β αSyn HOECHST
J
30DOD
60DOD
SNCA in nNOS+ voxels
SNCA in GABA+ voxels
SNCA in vGLUT2+ voxels
SNCA in NET+ voxels
SNCA in Serotonin+ voxels
SNCA in NPY+ voxels
SNCA in ChAT+ voxels
SNCA in S100β+ voxels
SNCA in TH+ voxels
SNCA in GFAP+ voxels
Ctrl_1
Ctrl_2
3xSNCA

Figure S6. Midbrain-hindbrain assembloid characterization. (A) Representative images of MO and HBO at 16 days of differentiation (DOD), a few days after embedding. The red line was used to delimitate the core of the organoid and calculate the area occupied by it. At this time-point, HBO area was already significantly higher to that of the MO, suggested by a Welch's t test with *p value = 0.0335. The panels on the right show representative pictures of fixed organoids at the end of the co-culture and after separation, highlighting the visible differences in size (2-3mm for HBO and 1-2mm for MO) and in morphology that permits their distinction at the moment of dissection. **(B)** Representative immunofluorescence images of an entire assembloid after retrograde viral tracing experiments. Whole-mount allowed the visualization of intrinsic RFP and GFP fluorescent expression from the viruses and the colocalization of the two signals on the assembloids (yellow). Scale bar = 200µm. **(C)** Image of the HBO side of an infected assembloid (different to the one shown in panel B) showing colocalization of the rabies virus (RFP) and neurons (MAP2). Scale bar = 50µm. Note that images in C and D are maximum intensity projections of a z stack acquisitions. **(D)** The cut-lines of MD to accommodate the MEA-well ensuring the positions of electrode array beneath the tunnels. **(E)** A schematic representation of the MD tunnels aligned with electrodes and the arrangement of the exposed four reference electrodes in the corners. **(F)** Representative brightfield image of an assembloid on MD MEA, where MO were placed on the left compartment and HBO on the right compartment, leaving connections between them to occur and be recorded in the microtunnels. Scale bar = 200µm.

Figure S6.

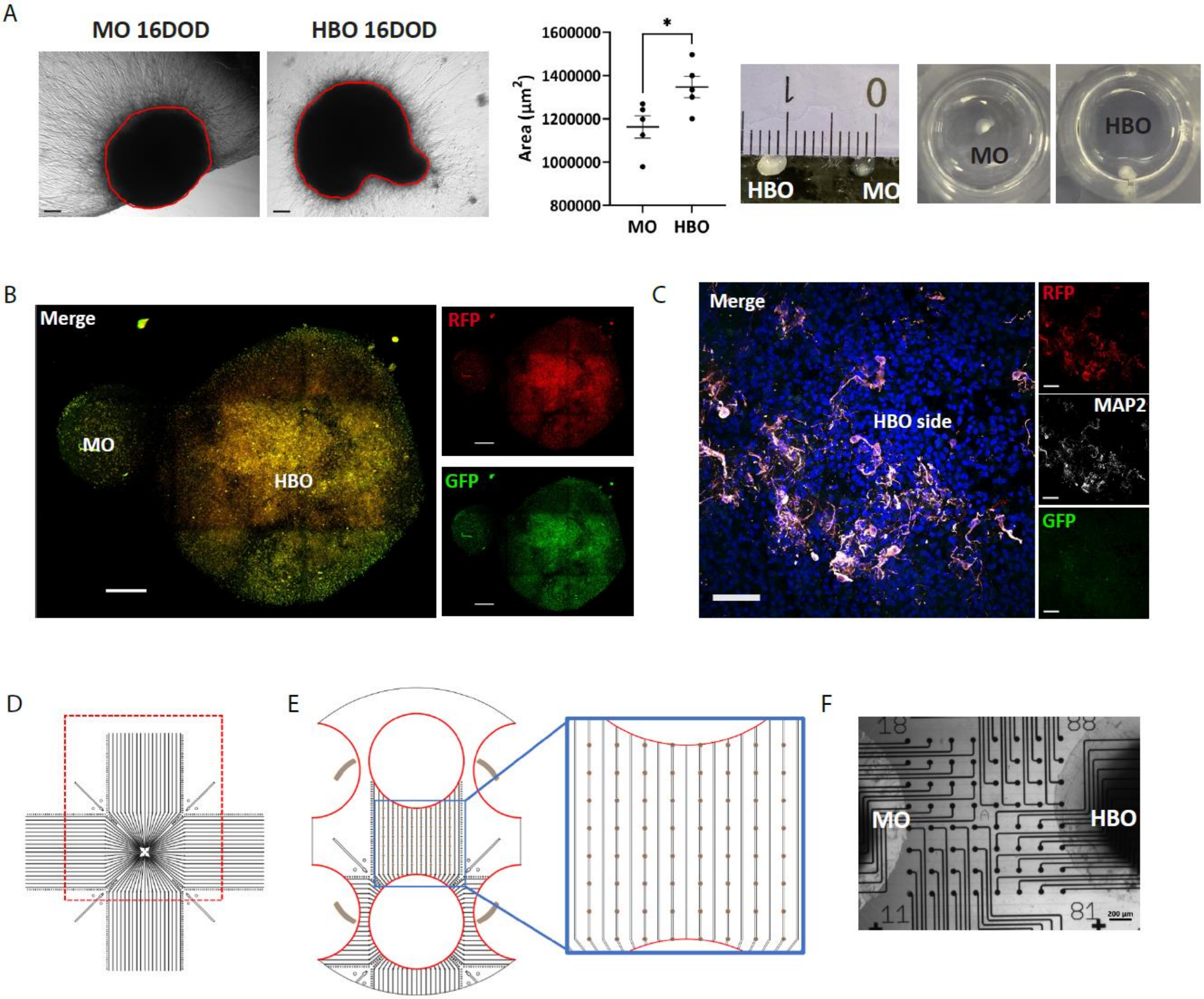
A
MO 16DOD
HBO 16DOD
*
Area (μm²)
1600000
1400000
1200000
1000000
800000
MO
HBO
HBO
MO
MO
HBO
B
Merge
MO
HBO
RFP
GFP
C
Merge
HBO side
RFP
MAP2
GFP
D
E
F
18
88
MO
HBO
11
81
200 μm

Figure S7. α-Syn pathology on healthy MO upon assembly with PD HBO. (A) UMAP plots from the different snRNAseq samples showing higher population diversity in HBO than MO models. On the right-hand panel the relative distribution (in percentage) of the different cluster populations per model is shown. **(B)** Representative images of pS129 staining in sections of MO after assembly. Zoomed in areas are delimited by a dashed squared area. Scale bar = 200µm. **(C)** Image analysis quantification shows a tendency towards increased pS129 staining levels on MO upon assembly with 3xSNCA HBO. Data resulted from at least 3 independent organoid batches. Data is shown as box plot where median is shown and was found not significant by Mann-Whitney test. **(D)** Image analysis quantification of non-assembled control MO after 30 and 60 days of differentiation showing no increase in α-Syn between the two time points, suggesting that the increase of α-Syn is associated to the assembly with PD HBO. Data resulted from at least 3 independent organoid batches. Data is represented as box plots with min and max bars. Statistical significance was assessed by Kolmogorov-Smirnov test: $^{**}p < 0.01$. **(E)** Levels of *SNCA* expression of across all snRNAseq models and throughout the different cluster cell types showing a significantly increased expression in healthy MO upon assembly with PD 3xSNCA HBO in neural progenitors, astrocytes, GABAergic neurons and dopaminergic progenitors from cluster number 2. DA stands for dopaminergic. **(F)** Representative images of TH, α-Syn and pS129 stainings in sections of healthy MO after being exposed to either healthy or PD 3xHBO media. Delimited areas are zommed in regions for better visualization. Scale bar = 200µm. **(G)** Quantification of stained images for α-Syn and pS129 showing significant increase in α-Syn in MO exposed to PD 3xSNCA HBO media, but no significant change in pS129 was observed. Data is shown as violin plots where median and quartiles are shown. Statistical significance was tested with a Kolmogorov-Smirnov test: $^{****}p < 0.0001$.

Figure S7.

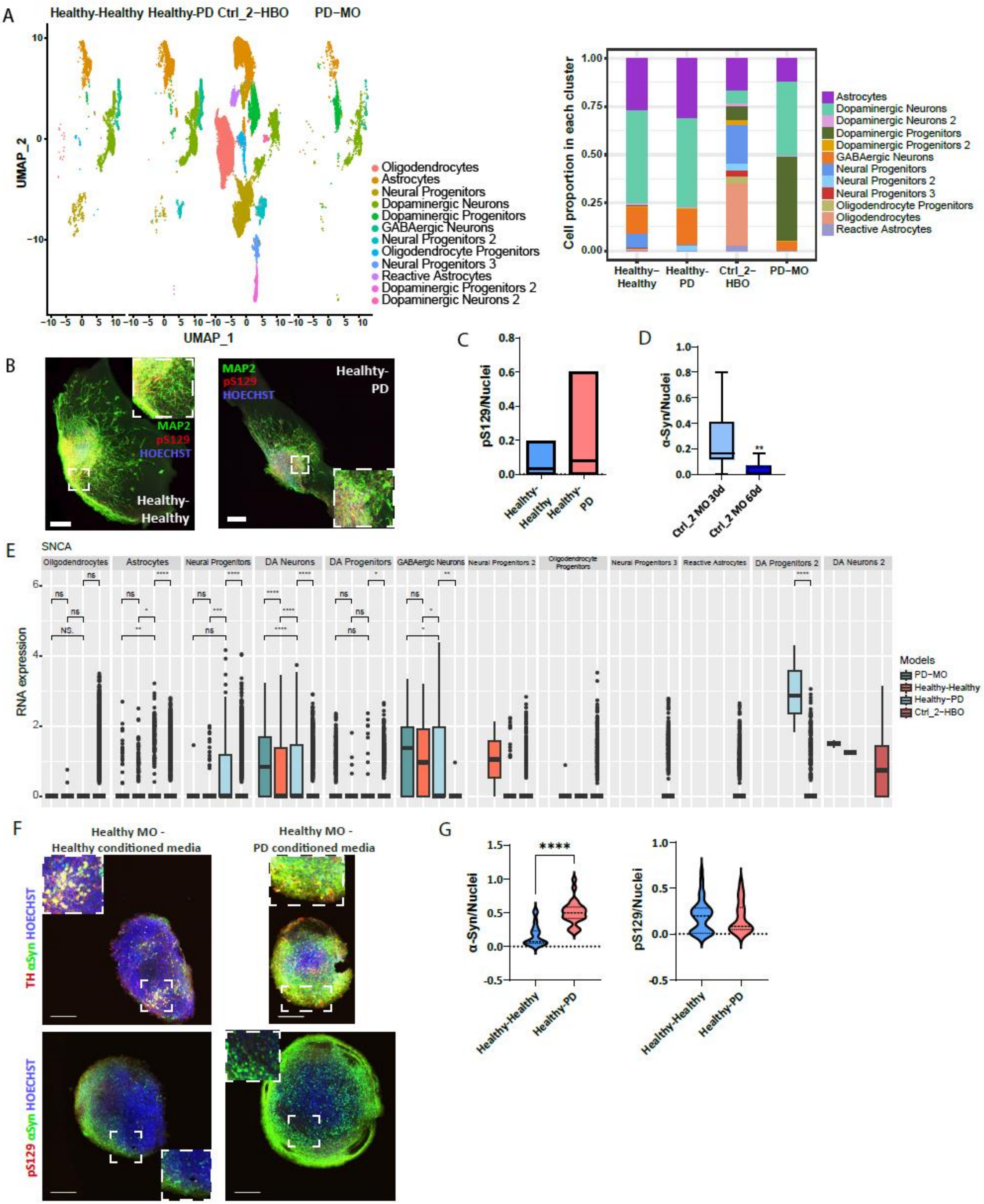
A
Healthy-Healthy Healthy-PD Ctrl_2-HBO PD-MO
UMAP_2
UMAP_1
Oligodendrocytes
Astrocytes
Neural Progenitors
Dopaminergic Neurons
Dopaminergic Progenitors
GABAergic Neurons
Neural Progenitors 2
Oligodendrocyte Progenitors
Neural Progenitors 3
Reactive Astrocytes
Dopaminergic Progenitors 2
Dopaminergic Neurons 2
Cell proportion in each cluster
Healthy-Healthy
Healthy-PD
Ctrl_2-HBO
PD-MO
Astrocytes
Dopaminergic Neurons
Dopaminergic Neurons 2
Dopaminergic Progenitors
Dopaminergic Progenitors 2
GABAergic Neurons
Neural Progenitors
Neural Progenitors 2
Neural Progenitors 3
Oligodendrocyte Progenitors
Oligodendrocytes
Reactive Astrocytes
B
MAP2
pS129
HOECHST
Healthy-Healthy
Healhty-PD
C
pS129/Nuclei
Healthy-Healthy
Healthy-PD
D
α-Syn/Nuclei
Ctrl_2 MO 30d
Ctrl_2 MO 60d
E
SNCA
Oligodendrocytes
Astrocytes
Neural Progenitors
DA Neurons
DA Progenitors
GABAergic Neurons
Neural Progenitors 2
Oligodendrocyte Progenitors
Neural Progenitors 3
Reactive Astrocytes
DA Progenitors 2
DA Neurons 2
RNA expression
Models
PD-MO
Healthy-Healthy
Healthy-PD
Ctrl_2-HBO
F
Healthy MO - Healthy conditioned media
Healthy MO - PD conditioned media
TH αSyn HOECHST
pS129 αSyn HOECHST
G
α-Syn/Nuclei
pS129/Nuclei
Healthy-Healthy
Healthy-PD

Figure S8. Synaptic alterations on healthy MO upon assembly with PD HBO. (A) Bar graph highlighting the most deregulated genes related to synapses, development, neurogenesis and synaptogenesis in the Healthy-PD assembloid condition. Note that most genes are upregulated in the case of healthy MO after assembly with 3xSNCA HBO. **(B)** Representative images of synaptic markers Synaptophysin and PSD-95 in MO after assembly. Squared area is enlarged on the right-hand side panel of every picture. Scale bars = 200μm. **(D)** Image analysis quantification of synaptic markers Synaptophysin and PSD-95 in MO sections after assembly. Data was collected from one section per organoid of at least 5 independent experiments. Data is shown as truncated violin plots, where the dashed line represents the median and the dotted line, the quartiles. If statistical significance is not shown, comparison was not significant by Mann-Whitney test. **(D)** Time course of mean firing rate and number of spikes of assembloids on the MD MEA over 50 days of assembly on the MEA. Please note that the Healthy-Healthy data is the same as shown in Figure 4 and Healthy-PD condition is added for comparison. Note that both conditions have similar behaviours, with the Healthy-PD condition having lower values in most time points. The data collection for the MD MEA experiment was performed for 2 independent MO and HBO batches, with 3 organoids per batch, which were placed within the same Axion plate. Data is represented as mean ± SD.

Figure S8.

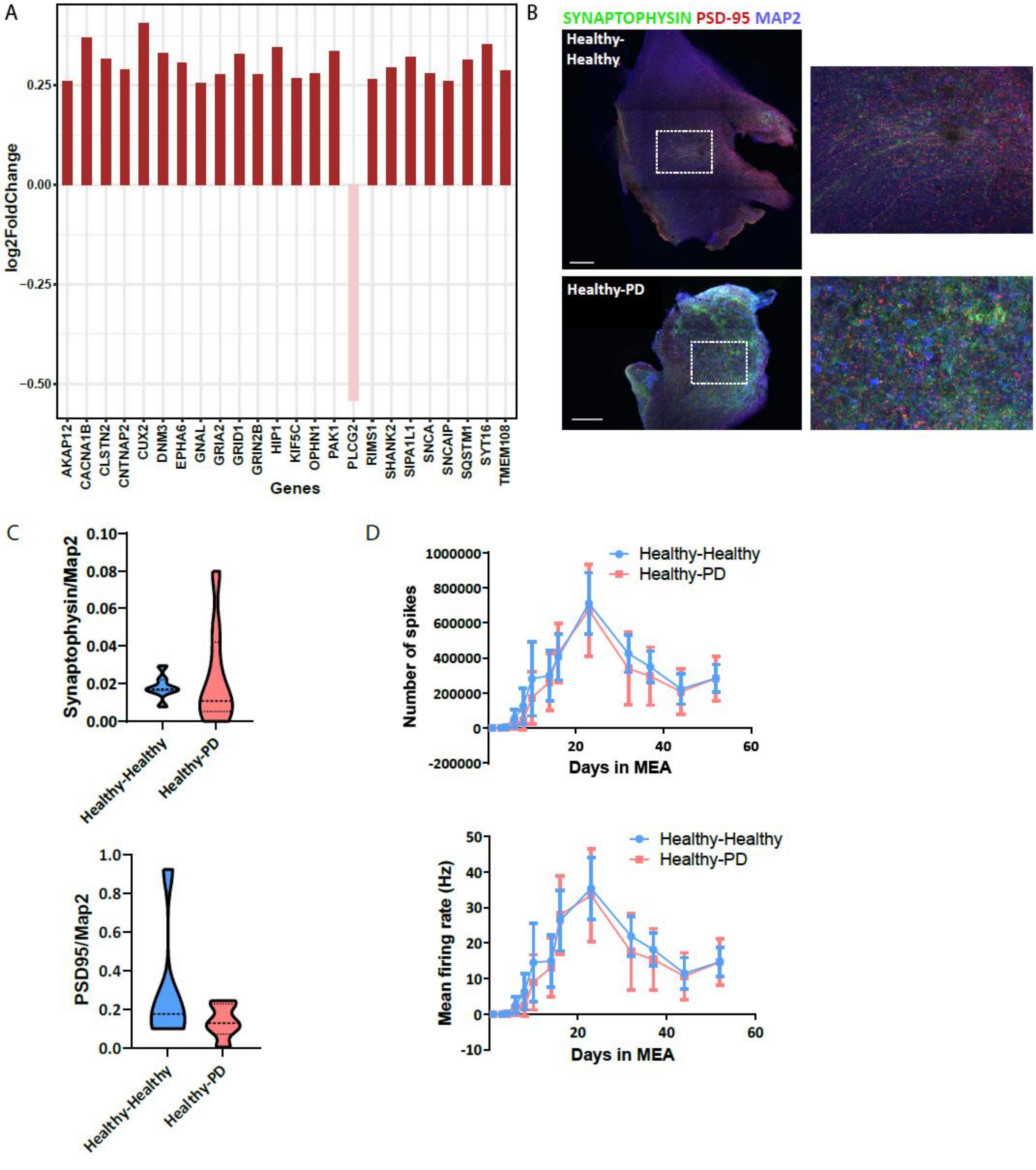
A
log2FoldChange
0.25
0.00
−0.25
−0.50
AKAP12
CACNA1B
CLSTN2
CNTNAP2
CUX2
DNM3
EPHA6
GNAL
GRIA2
GRID1
GRIN2B
HIP1
KIF5C
OPHN1
PAK1
PLCG2
RIMS1
SHANK2
SIPA1L1
SNCA
SNCAIP
SQSTM1
SYT16
TMEM108
Genes
B
SYNAPTOPHYSIN PSD-95 MAP2
Healthy-Healthy
Healthy-PD
C
Synaptophysin/Map2
0.10
0.08
0.06
0.04
0.02
0.00
Healthy-Healthy
Healthy-PD
PSD95/Map2
1.0
0.8
0.6
0.4
0.2
0.0
Healthy-Healthy
Healthy-PD
D
Number of spikes
1000000
800000
600000
400000
200000
0
-200000
Days in MEA
20
40
60
Healthy-Healthy
Healthy-PD
Mean firing rate (Hz)
50
40
30
20
10
0
-10
Days in MEA
20
40
60
Healthy-Healthy
Healthy-PD

Figure S9. Uncropped western blot membranes of this manuscript organized by order of figures. (A) Membrane corresponding to western in Figure 3C. We show overlay with the full marker. **(B)** Membrane corresponding to blot from Figure 3E. Green and red channels are overlayed. **(C)** Uncropped membrane corresponding to the display in Figure 5F. The membrane was incubated separately with either the synuclein antibodies or the housekeeping and neuronal marker. Hence, both exposures are shown. **(D)** Lanes 1, 2 and 3 correspond in this full membrane correspond to the ones showed in Figure S1B. The full membrane is shown in greyscale for better visualization, but fluorescent conjugated secondary antibodies were used. Therefore, the ladder was added in its original form. **(E)** Membrane corresponding to Figure S4G. The original molecular weight ladder was overlayed.

Figure S9.

A Uncropped membrane from Figure 3C

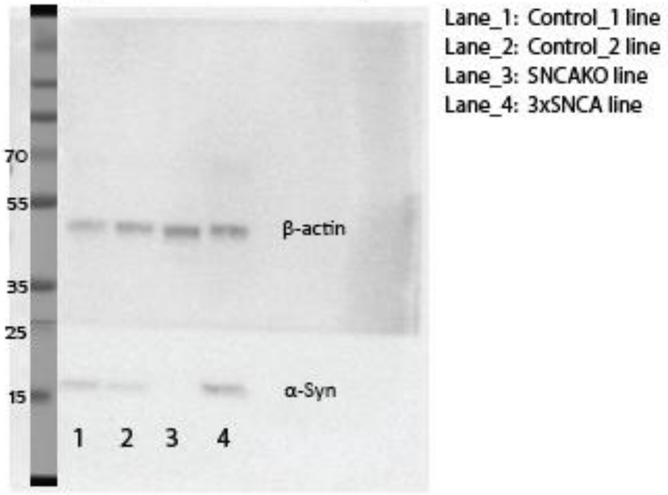

B Uncropped membrane from Figure 3E

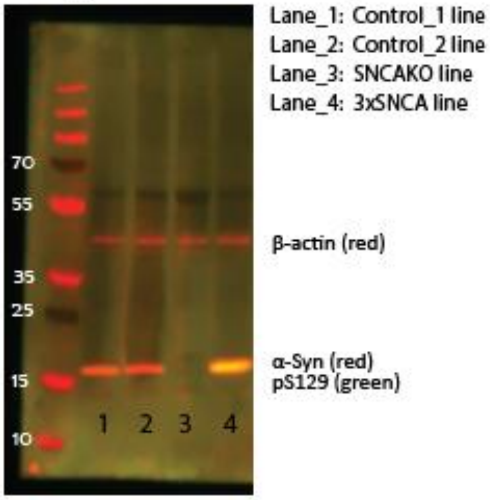

C Uncropped membrane from Figure 5F

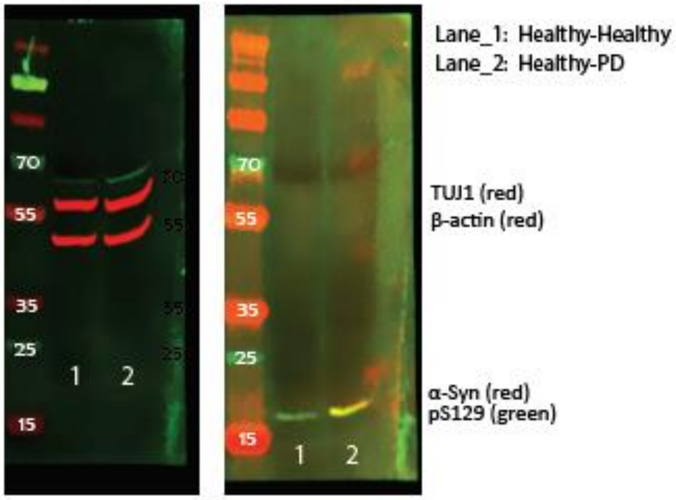

D Uncropped membrane from Figure S1B

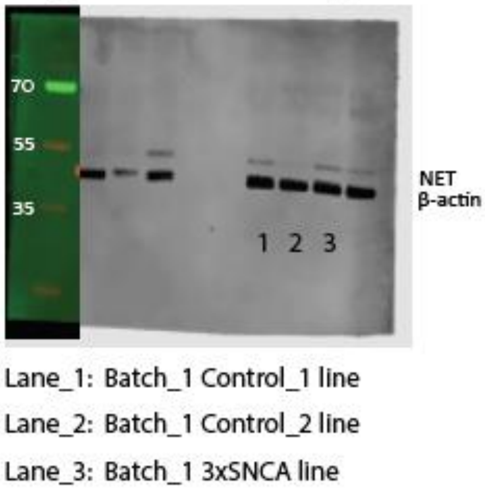

E Uncropped membrane from Figure S4G

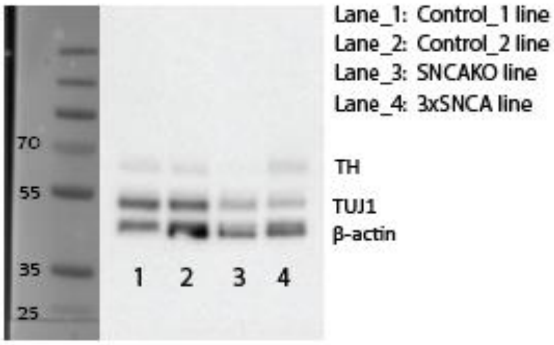

Supplementary Methods

Fabrication of microtunnel devices (MDs) for integration into microelectrode array well plates

The fabrication of MDs followed the procedures outlined by Bastiaens and colleagues[77]. To create the MDs mold, photolithography was employed using SU-8 2010 (Microchem Inc., Newton, MA, United States) on a 4-inch silicon wafer. The mold fabrication process began with the coating of a silicon wafer with resist, achieved by spinning it at 4500 rpm for 30 s, and subsequently performing a soft-bake at 95 ºC for 140 seconds on a hot plate (EchoTherm™, HS60 series). Next, the wafer was exposed to collimated UV light (IDONUS, UV-EXP 150R, Neuchatel, Switzerland) through a photomask (designed in AutoCAD 2022 and printed by CAD/Art Services Inc., Bandon, OR, United States) at a dose of 220 mJ/cm^2, with the photomask design available in Supplementary methods Figure. SM1b. Afterwards, a post-exposure bake lasting 3.5 minutes at 95 ºC was carried out on the same hot plate, followed by the development of the resist within a beaker containing developer (mrDev-600, Microresist Technology GmbH), and finalized with rinsing and drying. The thickness of the SU-8 structures was measured at 6 µm using a profilometer (Bruker Dektak XT, Massachusetts, USA). The completed mold was then placed in a 120 mm square petri dish (Greiner Bio-One International GmbH, the Netherlands) and stored at room temperature within a laboratory cabinet to protect it from potential external contaminants like dust until it was needed for future use.

To fabricate the PDMS MD via soft lithography utilizing the fabricated SU-8 mold, the PDMS elastomer and cross-linking agents (SYLGARD 184, Dow Corning, Midland, MI, USA) were mixed at a 10:1 ratio and subjected to vacuum degassing for 20 minutes. Subsequently, aluminum foil was wrapped around the 4-inch SU-8 mold Si wafer with an SU-8 mold to create a cavity for pouring a specific volume of PDMS onto the mold. We poured 20 gr of the PDMS mixture to achieve a PDMS thickness of 2.5 mm. The curing process took place at 65°C (in DL 53 oven, VWR®) for a duration of 4 h. Afterward, the PDMS was peeled, and holes for the cell culture reservoirs were incorporated with a 2-mm biopsy tool. We employed a stencil to accurately position the reservoirs in their designated locations. The Computer Aided Design (CAD) drawing of the stencil is depicted in Supplementary methods Figure. SM1c, and a detail instructions for fabrication of the stencil can be found in the next section.

Cell reservoir creation and PMMA stencil fabrication

After peeling off the PDMS from SU-8 mold, we created cell culture reservoirs by using a biopsy tool. This process was facilitated by employing a PMMA stencil to position the necessary reservoirs precisely. To ensure proper alignment, the central circle of the stencil was initially matched with the center of the MD under a stereomicroscope. Subsequently, the stencil was fixed with tape to prevent any movement during the punching process. We then utilized a 2-mm biopsy punch to generate the required reservoirs in the designated locations on the MD. The PMMA stencil was fabricated using a laser cutting equipment (Universal Laser System, VLS Model 3.50, ENGRAVING SYSTEMS, LLC, Connecticut, USA) from a 1-mm thick polymethyl methacrylate (PMMA) sheet (Precision acrylic glass, transparent, colorless, Modulor, Germany). Before using the fabricated PMMA stencil, it protective foil was removed. Subsequently, the stencil was cleaned in DI water and dried to eliminate any debris resulting from the laser cutting process on the PMMA. To fit the MD within the MEA-wells while containing the array of electrodes beneath the tunnels, we cut the MD as illustrated in Supplementary Figure S6D. One punch was placed in the center of the MD, and another punch was positioned at the end of the tunnels with a center-to-center distance of 3.4 mm. Additionally, four more punches were made to expose the reference electrodes. The layout of the MD aligned with MEA substrate is illustrated in Supplementary Figure S6E.

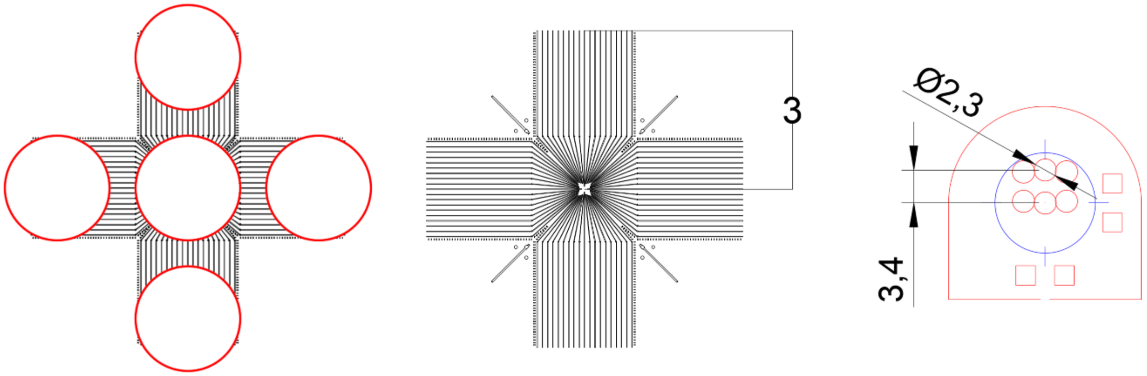

(a) **(b)** **(c)**

Fig. SM1. **(A)** The modified MD design layout, combining radial MD properties with compatibility for an electrode array. **(B)** The photomask design used to fabricate this MD configuration. **(C)** The layout of the PMMA stencil for integrating reservoirs into the MD.

Supplementary Tables

Table S1. Information of cell lines used in the present study.

**Internal cell line identifier**	**Cell line name**	**Origin (Healthy, WT; Patient, PD)**	**Sex (Male, M; Female, F)**	**Age of sampling**	**Age of onset**	**Source**	**Reference**	**Karyotype**
362	Ctrl1	WT	M	30	-	Coriell institute	GM25256*I	Normal
232	Ctrl2	WT	F	53	-	*Reinhardt et al., 2013*		Normal
336	3xSNCA	PD	F	55	50	EBISC	EDi001-A	Normal
320	SNCAKO	WT	M	67	-	*Barbuti et al.,* 2020		

Table S2. List of primers used for quantitative PCR.

Gene	Sequence	Sense	Amplicon size
LMX1B	ATCGTGGCCATGGAACAGAG	Forward	177bp
	GTCTGAGGAGCCGAGGAAG	Reverse	
LMX1A	CAACTCAACAGAGGCGAGCATT	Forward	128bp
	GTTTTGGAACCACACCTGGAC	Reverse	
EN1	AACCGCTACATCACGGAGCA	Forward	102bp
	GATCTTGGCGCGCTTGTTCT	Reverse	
ACTB	TCAAGATCATTGCTCCTCCTGAG	Forward	87bp
	ACATCTGCTGGAAGGTGGACA	Reverse	
HOXA2	CGTCGCTCGCTGAGTGCCTG	Forward	92bp
	TGTCGAGTGTGAAAGCGTCGAGG	Reverse	
NKX2.1	CGTACCAGGACACCATGAGG	Forward	168bp
	GGGCCATGTTCTTGCTCAC	Reverse	
NKX6.1	GCCTCGGAGAACGAGGAAGA	Forward	106bp
	CGCTGCTGGACTTGTGCTTC	Reverse	
GBX2	GTTCCCGCCGTCGCTGATGAT	Forward	118bp
	GCCGGTGTAGACGAAATGGCCG	Reverse	
NKX2.2	GAAGCGCCGAGTGCTCTTCTCC	Forward	375bp
	GCCGAGCTGTACTGGGCGTTGT	Reverse	
HOXB4	CTACTGCCGCTGCTGGAAGA	Forward	122bp
	TGTGTGTGTGTTACCGTGACCAA	Reverse	
PAX7	ACCCCTGCCTAACCACATC	Forward	121bp
	GCGGCAAAGAATCTTGGAGAC	Reverse	
HOXC4	GGCAGCTACCCCGGGTACT	Forward	101bp
	TGTGAGTTATGTTTTATAACCTGGTAATG TC	Reverse	
OTX2	GCCAATCCTTGGTTGAATCTTAGG	Forward	120bp
	CAATCAGTCACACAATTCACACAGC	Reverse	
PAX6	CCAGGGCAATCGGTGGTAGT	Forward	84bp
	ACGGGCACTCCCGCTTATAC	Reverse	
RPL37A	GTGGTTCCTGCATGAAGACAGTG	Forward	84bp
RPL37A	TTCTGATGGCGGACTTTACCG	Reverse	

Table S3. Antibody list and application.

Antibody	Species	Concentration	Source	Catalog number	RRID	Used in
GABA	Guinea pig	1:400	Abcam	ab17413	AB_443865	Immunostaining
GAD67	Mouse	1:400	Sigma	G5419	AB_261978	Immunostaining
Serotonin	Rat	1:100	Millipore	MAB352	AB_11213564	Immunostaining
TPH2	Rabbit	1:500	Abcam	ab111828	AB_10862137	Immunostaining
Tyrosine Hydroxylase	Rabbit	1:1000	Abcam	ab112	AB_297840	Immunostaining
Tyrosine Hydroxylase	Mouse	1:600	Millipore	MAB5280	AB_2201526	Immunostaining
Tyrosine Hydroxylase	Chicken	1:500	Abcam	ab76442	AB_1524535	Immunostaining
MAP2	Chicken	1:1000	Abcam	ab5392	AB_2138153	Immunostaining
MAP2	Rabbit	1:100	Abcam	ab32454	AB_776174	Immunostaining
ChAT	Rabbit	1:100	Thermo Fisher Scientific	PA5-29653	AB_2547128	Immunostaining
OTX2	Goat	1:200	Neuromics	GT15095	AB_2157174	Immunostaining
GBX2	Rabbit	1:500	Thermo Fisher Scientific	PA5-66953	AB_2662957	Immunostaining
vGLUT2	Mouse	1:600	Abcam	ab79157	AB_1603114	Immunostaining
NET	Rabbit	1:500	NOVUS Biologicals	NBP1-60120	AB_11014401	Immunostaining/ Western Blot
nNOS	Guinea pig	1:500	Synaptic Systems	432 005	AB_2832223	Immunostaining
GFAP	Chicken	1:1000	Millipore	AB5541	AB_177521	Immunostaining
S-100 (beta-subunit)	Mouse	1:1000	Sigma	S2532	AB_477499	Immunostaining
Neuropeptide Y	Chicken	1:500	Synaptic Systems	394 006	AB_2814940	Immunostaining
α-Synuclein (2A7)	Mouse	1:1000	NOVUS Biologicals	NBP1-05194	AB_1555287	Immunostaining
Neurexin-1	Goat	1:200	Abcam	ab77596	AB_1566476	Immunostaining
Neuroligin-2	Rabbit	1:200	Synaptic Systems	129 203	AB_993014	Immunostaining
Synaptophysin	Mouse	1:50	Abcam	ab8049	AB_2198854	Immunostaining
PSD-95	Rabbit	1:100	Invitrogen	51-6900	AB_2533914	Immunostaining
α-Synuclein (211)	Rabbit	1:1000	Santa Cruz	sc-12767	AB_628318	Western Blot

α-Synuclein [MJFR1]	Rabbit	1:1000	Abcam	ab138501	AB_2537217	Dot Blot/ Immunostaining
Secondary AB Anti- rabbit IgG ECL	Donkey	1:1000	GE Healthcare	NA934	AB_772206	Dot Blot
Phospho- α-Synuclein (Ser129) (D1R1R)	Rabbit	1:500	Cell Signaling Technology	23706S	AB_2798868	Immunostaining/ Western Blot
ß-actin	Mouse	1:10000	Cell Signaling Technology	3700	AB_2242334	Western Blot
Tyrosine Hydroxylase TH (H-196)	Rabbit	1:1000	Santa Cruz	sc-14007	AB_671397	Western Blot
ßIII Tubulin	Mouse	1:20000	BioLegend	801201	AB_2313773	Western Blot
Anti-chicken 488	Goat	1:1000	Invitrogen	A-11039	AB_142924	Immunostaining
Anti-chicken 488	Donkey	1:1000	Jackson Immuno research	703-545-155	AB_2340375	Immunostaining
Anti-goat 488	Donkey	1:1000	Invitrogen	A-11055	AB_2534102	Immunostaining
Anti-rabbit 488	Donkey	1:1000	Invitrogen	A21206	AB_2535792	Immunostaining
Anti-chicken 568	Goat	1:1000	Invitrogen	A-11041	AB_2534098	Immunostaining
Anti-chicken 568	Donkey	1:1000	Thermo	A78950	AB_2921072	Immunostaining
Anti-rabbit 568	Goat	1:1000	Invitrogen	A11036	AB_2534094	Immunostaining
Anti-rabbit 568	Donkey	1:1000	Invitrogen	A10042	AB_2534017	Immunostaining
Anti-rabbit 647	Goat	1:1000	Invitrogen	A-21244	AB_141663	Immunostaining
Anti-mouse IgG1 647	Goat	1:1000	Invitrogen	A-21240	AB_2535809	Immunostaining
Anti-mouse IgG2a 647	Goat	1:1000	Invitrogen	A-21241	AB_2535810	Immunostaining
Anti-rat 568	Goat	1:1000	Invitrogen	A-11077	AB_2534121	Immunostaining
Anti-goat 647	Donkey	1:1000	Invitrogen	A21447	AB_141844	Immunostaining
Anti-guinea pig 647	Goat	1:1000	Invitrogen	A21450	AB_141882	Immunostaining
Anti-rabbit 647	Donkey	1:1000	Invitrogen	A31573	AB_2536183	Immunostaining
Anti-mouse 647	Donkey	1:1000	Invitrogen	A31571	AB_162542	Immunostaining

Table S4. Percentages of different cell populations within the different models: midbrain organoids after assembly (Healthy-Healthy, Healthy-PD), Ctrl_2-HBO model and PD-MO model, considered in the snRNAseq dataset.

Cell types	Healthy-Healthy (%)	Healthy-PD (%)	Ctrl_2-HBO (%)	PD-MO (%)
Oligodendrocytes	0.733390854	0.016641704	33.27364439	0.063572791
Astrocytes	27.04918033	31.1699118	16.85498108	12.39669421
Neural Progenitors	7.722174288	0.93193543	20.81715006	0.445009536
Dopaminergic Neurons	48.66264021	46.19737061	6.625472888	38.33439288
Dopaminergic Progenitors	0.992234685	0.798801797	7.41740227	43.99237127
GABAergic Neurons	13.89128559	18.48893327	0.035308953	4.640813732
Neural Progenitors 2	0.086281277	2.313196871	3.442622951	0
Oligodendrocyte Progenitors	0.819672131	0.033283408	3.286254729	0
Neural Progenitors 3	0	0	2.549810845	0
Reactive Astrocytes	0	0	2.292559899	0
Dopaminergic Progenitors 2	0	0.049925112	2.14627995	0
Dopaminergic Neurons 2	0.043140638	0	1.25851198	0.127145582
